# Impact of Swine and Cattle Manure Treatment on the Microbial Composition and Resistome of Soil and Drainage Water

**DOI:** 10.3390/microorganisms11010017

**Published:** 2022-12-21

**Authors:** Phil Colgan, Elizabeth L. Rieke, Khurram Nadeem, Thomas B. Moorman, Michelle L. Soupir, Adina Howe, Nicole Ricker

**Affiliations:** 1 Department of Agricultural and Biosystems Engineering, Iowa State University, Ames, IA 50011, USA; 2 Department of Math and Statistics, University of Guelph, Guelph, ON N1G 2W1, Canada; 3 National Laboratory for Agriculture and the Environment, United States Department of Agriculture Agricultural Research Service, Ames, IA 50011, USA; 4 Department of Pathobiology, Ontario Veterinary College, University of Guelph, Guelph, ON N1G 2W1, Canada

**Keywords:** antimicrobial resistance, microfluidic qPCR, soil, swine manure, cattle manure, drainage water

## Abstract

Evaluating potential environmental and clinical impacts of industrial antibiotic use is critical in mitigating the spread of antimicrobial resistance. Using soil columns to simulate field application of swine or cattle manure and subsequent rain events, and a targeted qPCR-based approach, we tracked resistance genes from source manures and identified important differences in antimicrobial resistance gene transport and enrichment over time in the soil and water of artificially drained cropland. The source manures had distinct microbial community and resistance gene profiles, and these differences were also reflected in the soil columns after manure application. Antibiotic resistance genes (ARGs) were only significantly enriched in effluent samples following the first rain event (day 11) for both soil types compared to the control columns, illustrating the high background level of resistance present in the control soils chosen. For swine, the genes *tetQ*, *tet(36)*, *tet44*, *tetM*, *sul2* and *ant(6)-ib* persisted in the soil columns, whereas *tetO*, *strB* and *sul1* persisted in effluent samples. Conversely, for cattle manure *sul2* and *strB* persisted in both soil and effluent. The distinct temporal dynamics of ARG distribution between soil and effluent water for each manure type can be used to inform potential mitigation strategies in the future.

## 1. Introduction

Antimicrobial resistance (AMR) is one of the largest human health concerns of this generation, as the continued emergence of resistance threatens to interfere with our ability to treat and prevent human infectious diseases [1]. Combating AMR requires a One Health approach, meaning that the research and policies developed should take a holistic approach that acknowledges the complexity of AMR emergence and spread within human, animal and environmental reservoirs [2,3,4]. The role of animal production as a hotspot for AMR emergence is widely accepted, and dissemination of AMR bacteria and genes to the environment after field application of manure is an area of active research [5,6,7,8,9].

Antimicrobial use decisions in the livestock industry have been shown to impact the abundance of antibiotic resistant bacteria (ARB) and antibiotic resistance genes (ARGs), commonly referred to as the resistome, in the gut microbial communities and feces of these animals [10,11,12,13,14]. The usage of antibiotics in animal production also impacts the resistome of soil and water within integrated agricultural systems [2]. Manure collected from antibiotic-treated cattle and swine is often used to fertilize crop soil. When the manure is applied to soil, ARGs and residual antibiotics from manure are introduced into the soil and ARB already present in the soil can be enriched within the soil microbial community in response to the nutrient additions from the manure [12,15,16,17,18,19,20]. The potential for transport of manure-associated ARBs and ARGs from the soil and into the surrounding environment is enhanced by artificial subsurface drainage systems. These drainage systems utilize a series of corrugated drainage pipes installed underground to divert excess water from the soil and into adjacent rivers and streams, in order to create a more hospitable root environment for cropping systems. Over 20 million hectares of land in the United States contain artificial drainage systems, including over 3000 drainage districts in the state of Iowa alone [21]. ARBs and ARGs are transported from manure-treated soil into subsurface drainage systems [18] that lead to adjacent water sources where they may spread throughout the environment with the potential for contaminating food and water supplies [22].

The risks of ARB and ARG transport after manure application are dependent on many factors that are different across the animal sectors. In addition to differences in fecal microbial communities between livestock species, antimicrobial use policies and practices have been shown to impact the specific resistance genes that will be most prevalent in the manure [18,23,24]. Different management strategies, including composting of manure and storage time prior to application, have been shown to influence antibiotic residues and resistant bacteria within the final applied product [7,25,26,27,28,29]. In addition, movement of ARB and ARG through the soil can also be impacted by the nature of the manure itself; for example, the dissemination of organisms after field application can be expected to differ based on the high-water content of swine manure compared to cattle manure. Each of these factors can be expected to impact the overall risk of manure application to downstream catchments, and for this reason we evaluated both swine and cattle manure in the current study.

The objective of this study was to identify differences in ARG transport and enrichment in the soil and drainage water of our model system over time following swine and cattle manure amendment. We designed a controlled experiment using soil columns and simulated rainfall events to collect soil and water samples from columns treated with cattle manure, swine manure, or no manure at defined intervals. In our previous studies, we have examined the impact of swine manure on soil communities with and without a history of manure application and identified persistent resistance genes using shotgun metagenomics [18]. Here we have extended this previous work to look at cattle and swine manure transport through soils that have a history of manure application, in order to understand the fluctuations in resistance gene abundance with manure application and the long-term impacts on the soil community. We applied microfluidic qPCR (MF-qPCR) for resistance gene monitoring [30] and have included specific mobile element targets associated with the genes identified in our previous studies to expand on our understanding of manure impacts on the soil community. We hypothesized that (1) soil columns amended with each manure type will be distinguished by unique changes in microbial community profiles and will have distinct abundances of ARGs that will be observable in manure-treated soil and drainage water samples, and that (2) the movement of ARGs through our model system into the effluent water will be different for each manure type.

## 2. Materials and Methods

### 2.1. Experimental Design

A total of 48 soil columns were collected from two crop fields within the Black Hawk Lake watershed on 7 May 2018 using a Giddings soil probe and 15.24 cm inner diameter, 61 cm long PVC pipes. The methods used for column construction and simulated rainfall events were described previously [31]. Briefly, soil probes were gently pushed into the soil to minimize disturbance to the soil column and foam blocks were used to stabilize the columns for transport. End caps with a drainage tube and fiberglass mesh were fitted to the bottom of the columns and filled with ASTM 20–30 Test Sand to form a soil–sand interface (Supplementary Figure S1). Twenty-four columns were collected from each field site, one with a history of cattle manure application and one with a history of swine manure application. Soil columns with a previous history of cattle manure application as well as cattle manure utilized in this work were collected from the Allee Demonstration Farm associated with Iowa State University. Soil columns were also collected from a swine farm within the same geographic area to minimize differences in soil properties between the types of columns. The swine manure was sourced from a separate research farm affiliated with Iowa State University. Both fields were cropped in corn-soybean rotations and utilize tile drainage with pipes approximately 1 m below ground level. Soils at both sites consisted of well to poorly drained Clarion loam, Nicollet clay loam, and Canisteo clay loam, with slopes ranging from 0 to 5%.

Manure application occurred on 29 June 2018 and was designed to mimic field application rates and practices (surface spread for cattle and knife injection for swine). Manure (~180 g, estimated based on an average estimated application rate of 10,000 gallons per acre) was applied to 12 columns from each site (swine or cattle manure history), while 12 columns from each site received only water and served as controls. Columns were kept at room temperature and were monitored at defined intervals for 145 days. Simulated rainfall events were performed by adding 1 L deionized water to each column (equivalent to 5.5 cm rainfall per column) on day 11, 33, 61, 82, 110 and 138 after manure application, using caps fitted with hypodermic needles as previously described [31]. Evaporation was estimated based on water loss from an open pan and equivalent water volume was added to the columns between rain events to maintain moisture content. Soil samples were collected on days 0 (prior to manure application, as controls), 17, 89, and 145 days after manure application by destructively sacrificing three columns and sampling from three locations after homogenizing the soil from the column. All water samples were filtered onto 0.22 um sterile filters and frozen under −80 °C until DNA extractions were performed with the MagAttract PowerWater DNA EP kit (Qiagen, Germantown, MD, USA). All soil samples were collected and subsampled for DNA extractions using the Qiagen MagAttract PowerSoil DNA EP kit (Qiagen, Germantown, MD, USA). The concentration of DNA was quantified on a Synergy HT plate reader with Quant-iT dsDNA Assay kit (Fisher, Rochester, NY, USA).

### 2.2. ARG Target Selection

The choice of ARGs used for screening in this study was based on their observed presence in swine and cattle manure metagenomes and their absence or limited detection in soil metagenomes. Shotgun metagenomes were generated using DNA extracted from 3 technical replicates each of swine and cattle manure as well as 3 biological replicates each of swine and cattle control soil. Libraries were prepared and sequenced at Iowa State University DNA Facility using an Illumina HiSeq 3000 platform (Illumina, San Diego, CA, USA) with 150 bp paired-end sequencing. All metagenomes were deposited in NCBI SRA accession number PRJNA662623. Reads were annotated against ARGs in the Comprehensive Antibiotic Resistance Database (CARD, version 2.0.1). ARGs that were abundant in manure metagenomes (defined as a minimum of 50 reads in all three biological replicates) and less than 10 reads in soil metagenomes, were selected as targets for MF-qPCR. In addition, targets from a previously published set of ARG primers [30] were also included along with the *tet(33)* gene previously identified in swine manure-treated soil and water metagenomes [18]. In total, 36 ARGs were targeted for this study as well as 6 mobile genetic elements (Supplementary Table S1).

### 2.3. 16S rRNA Microbial Community Analysis

Drainage water (50 mL) was filtered with a 0.22 μm sterile filter and frozen at −20 °C until extractions were performed using the MoBio PowerWater DNA extraction kits. Soil samples (0.25 g) were also frozen at −20 °C and then extracted using the PowerSoil 96-well Soil DNA isolation kit and normalized to a concentration of 10 ng/μL. Sequencing of the 16S rRNA was performed using the F515/R806 primers targeting the V4 region, MiSeq Reagent Kit v2 library preparation, and sequencing on an Illumina MiSeq sequencer following established protocols through the Iowa State University genomics center. Reads were analyzed using the DADA2 pipeline [32] with default settings. Samples with less than 4000 reads were removed from the analysis (*n* = 6 for cattle (including one manure), *n* = 1 for swine). Rare amplicon sequence variants (ASVs) were removed using a prevalence cut-off of 0.05% occurrence across all samples. Statistical analysis, linear regression conversion to absolute abundance and differential abundance were determined using Analysis of Compositions of Microbiomes with Bias Correction (ANCOM-BC) [33] with alpha set at 0.05 and Holm correction for multiple testing. The effect of day was modeled using a cubic smoothing spline fit with day as a numeric factor and day-treatment as an interaction effect.

### 2.4. Microfluidic Quantitative-PCR (MF-qPCR)

MF-qPCR assays were run on 9 separate 96.96 Fluidigm Dynamic Array Integrated Fluidic Circuits (IFCs) according to the manufacturer’s Evagreen protocol without pre-amplification. Six technical replicates of an inter-plate calibrator sample consisting of 10 randomly selected and pooled manure, soil and water samples (Supplementary Table S2) were included on each of the nine 96.96 IFCs for the purpose of calculating the variation within and between plates. Standard curves were generated for every primer set using 4 technical replicates of 10-fold dilutions of synthetic oligonucleotide standards ranging from 10^−6^ to 10^−2^ ng/uL. Standards were synthesized using gBlock Gene Fragments (Integrated DNA Technologies, Coralville, IA, USA) (Supplementary Table S3). The DNA samples screened with MF-qPCR include 6 biological replicates of each water sample, 6 biological replicates of day 0 control soil samples, 3 biological replicates of all other soil samples, and 3 technical replicates of each manure type. Each biological replicate consists of pooled DNA from 3 technical replicates. All of the samples and standards were amplified in duplicate to give an additional two technical replicates (Supplementary Table S4). All plates were loaded using a HX IFC Controller (Fluidigm, San Francisco, CA, USA) and placed in a BioMark HD (Fluidigm, San Francisco, CA, USA) for thermal cycling at 95 °C for 1 min, 30 cycles at 96 °C for 5 s and 60 °C for 20 s followed by melt curve analysis for 60–95 °C at a ramp of 1 °C/3 s.

Data were exported using the Real-Time PCR Analysis software, version 4.12 (Fluidigm, San Francisco, CA, USA) with the default peak sensitivity set to 7, peak ratio threshold of 0.7, melt temperature (T_m_) ranges individually set based on peaks observed in standards, quality threshold of 0.65 and linear baseline correction. The data were then processed and analyzed using RStudio, version 1.2.5001. Data points were discarded if duplicate technical replicates differed by more than 3 Ct and an upper Ct cutoff value of 28 was used as the limit of detection, resulting in 3340 total non-zero data points. Copy numbers were calculated using the respective standard curve for each gene and were then normalized by the copy number of the 16S rRNA gene amplified in each sample by the primer set 16S_Eub_338F_515R. Interplate and intraplate variance in calibrator samples were determined by calculating the standard error of Ct values for each gene between plates and within replicates on the same plate. Mann–Whitney U test was used to calculate significant differences (*p* < 0.05) in mean abundance between manure types and between manure-treated and corresponding control samples.

### 2.5. Enumeration of Fecal Indicator Bacteria

In order to compare genomic methods to traditional manure monitoring methods using Fecal Indicator Bacteria (FIB), counts of total Enterococci as well as antibiotic resistant Enterococci were obtained. For each column, 50 mL of drainage water was filtered on a 0.22 μm sterile filter and the filter was plated onto mEnterococcus (mE) agar (BD Difco) at 37 °C for 48 h following each rain event. Separate filtrates were used for mE without antibiotics as well as mE supplemented with tetracycline (16 mg/L) or tylosin (35 mg/L) to look for resistant isolates. On column destruction days, soil was diluted 1:10 (*w*/*v*) in water prior to direct plating of dilutions on mE with and without antibiotics, and CFU counts were adjusted to total counts per dry weight of soil. Total FIB from source manures were determined by the same protocol as soil samples.

## 3. Results

Soil columns were set up in parallel for swine and cattle manure experiments (see Section 2) and were obtained from fields with a history of swine or cattle manure application, respectively. For each experiment, columns were separated into treatment (manure application) or control (no manure) and all columns underwent simulated rain events on days 11, 33, 61, 82, 110 and 138. Water was collected from the bottom of the column following each rain event and destructive sampling was performed for soil collections on days 17, 89 and 145 post-treatment.

### 3.1. Changes in Bacterial Communities in Manure-Treated Soil

#### 3.1.1. Bacterial Composition in Manure

Cattle and swine source manure were both dominated by members of the *Firmicutes* and *Bacteroides* phyla, with significantly greater abundances of both taxa in swine manure than in cattle manure (adjusted *p*-values 4 × 10^−6^ and 0.005, respectively). The two manures could also be distinguished by the abundance of *Euryarchaeota*, *Verrucomicrobia*, *Deferribacteria*, *Cyanobacteria* and *Patescibacteria* in swine manure and the abundance of *Chloroflexi*, *Deinococcus-Thermus*, *Actinobacteria* and *Fibrobacteres* in cattle manure (Supplementary Figure S2A). The soil *Proteobacteria* communities prior to manure addition were similar for both the swine and cattle soils and were dominated by *Sphingomonadales*, *Rhizobiales*, and members of the *β-Proteobacteriales*. The cattle source manure also had significant levels of these taxa but was dominated by *Pseudomonadales*. Conversely, the swine source manure was quite dissimilar from the soil composition and was dominated by *Pseudomonadales*, *Desulfovibrionales*, *Cardiobacteriales* and *Aeromonadales* (Supplementary Figure S2B,C). Due to the differing bacterial compositions in the two manure types, the soil columns for each manure type and their respective controls were analyzed separately.

#### 3.1.2. Impact of Swine Manure on Microbial Composition in Soil and Column Effluent

**Figure 1:**
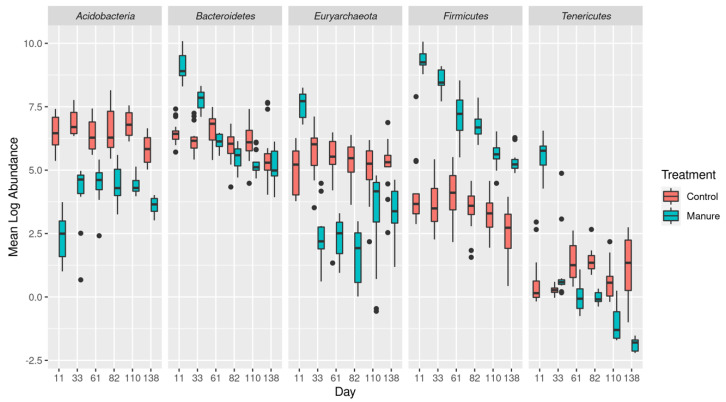
Impact of swine manure application on abundance of 5 key phyla in effluent water beginning on day 11 (after the first simulated rain event). Abundances have been natural log transformed and converted to absolute abundance (see Section 2). All associated *p*-values can be found in the Supplementary Materials.

The impact of swine manure on the taxonomic composition of effluent water was evident after manure addition and corresponded with increases in members of the *Firmicutes* and *Bacteroides* phyla which was consistent with the composition of the source manure. The impact of swine manure was still evident in the effluent on day 138 after manure treatment (Figure 1, Supplementary Table S5), with *Firmicutes* showing a greater than 4-log fold increase in the water from treated columns compared to the control columns. None of the other phyla that were significantly increased on day 11 showed consistent increases throughout the experiment, and *Bacteroidetes* was not significantly increased in the effluent by day 61 post-treatment. It is notable that the *Acidobacteria* were significantly decreased on day 11 after treatment compared to later days, and conversely, the *Euryarchaeota* and *Tenericutes* were increased on day 11 before decreasing significantly. The addition of manure had a smaller effect on the taxonomic composition of soil samples, as only *Firmicutes* and *Euryarchaeota* showed significantly impact from manure treatment (Supplementary Table S6).

#### 3.1.3. Impact of Cattle Manure Application on Soil Bacterial Composition and Column Effluent

**Figure 2:**
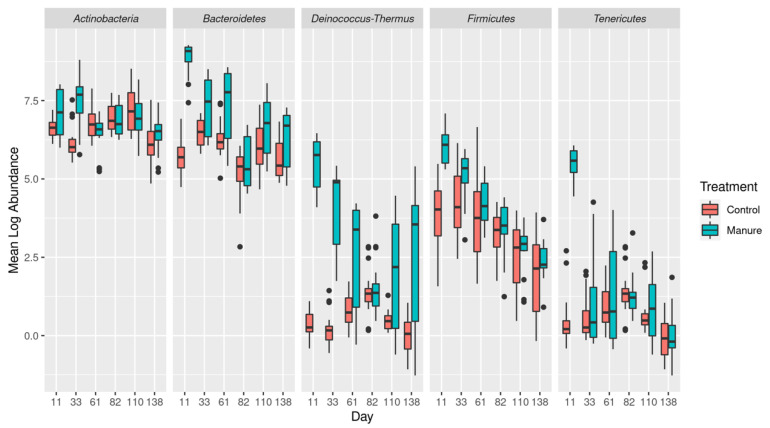
Impact of cattle manure application on abundance of 5 key bacterial phyla in effluent water beginning on day 11 (after the first simulated rain event). Abundances have been natural log transformed and converted to absolute abundance (see Section 2). All associated *p*-values can be found in the Supplementary Materials.

Effluent from cattle manure-treated columns showed elevated levels of *Deinococcus-Thermus* and *Tenericutes* in addition to *Bacteroidetes* and *Firmicutes* on day 11 after manure application (Figure 2, Supplementary Table S7). By day 33, *Deinococcus-Thermus* maintained a greater than 2-fold log increase over the control columns, and *Actinobacteria* were significantly increased. Though variable, *Deinococcus-Thermus* continued to be increased by the end of the study (day 138). Contrary to the effluent, the soil samples were only minimally impacted by manure application, with *Firmicutes* and *Deinococus-Thermus* increased only on day 17 and no other taxa showing strong increases after manure application (Supplementary Figure S3).

#### 3.1.4. Detection of Fecal Indicator Bacteria

Swine manure contained 7.1 × 10^6^ enterococci CFU g^−1^_dry_, with 74% resistant to tetracycline and 36% resistant to tylosin, while cattle manure contained 9.7 × 10^6^ enterococci CFU g^−1^_dry_, with less than one percent resistant to tetracycline and 38% resistant to tylosin. Average enterococci abundance in effluent from swine manure-treated soil columns 10 days after manure application were greater than 10^4^ CFU 100 mL^−1^, with 100% resistant to tylosin and tetracycline. Enterococci abundance decreased in effluent from swine manure-treated soil columns on day 33 with averages >10^2^ CFU 100 mL^−1^. No tylosin-resistant enterococci were detected on day 33, but 42% of enterococci remained resistant to tetracycline. No enterococci were detected in the effluent from swine manure-treated columns as of day 61 after manure application.

**Figure 3:**
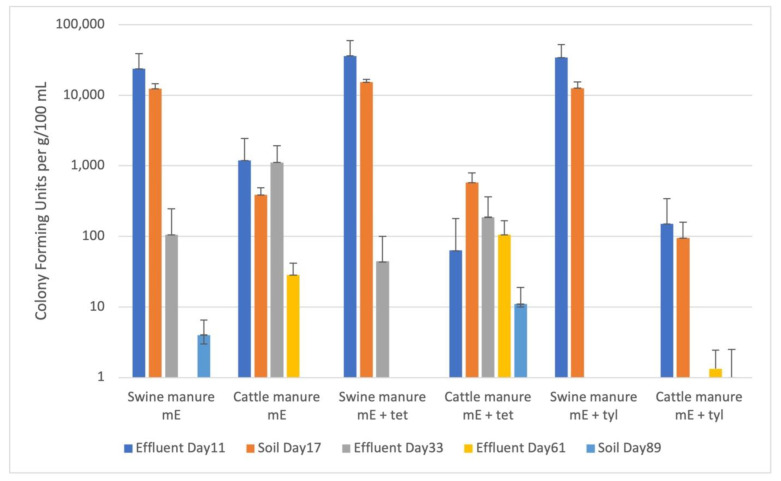
Abundance of enterococci recovered from effluent and soil of manure-treated columns over time. Recovery was performed on mEnterococcus media (mE) alone or with the addition of tetracycline (16 mg/L) or tylosin (35 mg/L) to look for resistant isolates. Abundance values are listed in CFU/g dry weight for soil samples or CFU/100 mL for effluent. Note that the y-axis is logarithmic scale. Whiskers represent standard deviation of CFU counts.

Enterococci abundance in effluent derived from soil columns treated with cattle manure were an order of magnitude lower than the swine manure-treated column effluent on day 10 after manure application (Figure 3). Additionally, the effluent contained much lower percentages of resistant enterococci with only 5% resistant to tetracycline and 13% resistant to tylosin. Enterococci concentrations in cattle manure-treated effluent at day 33 were consistent with concentrations at day 10, with averages greater than 10^3^ CFU 100 mL^−1^. No tylosin-resistant enterococci were present in effluent from cattle manure-treated columns at day 33; however, 17% of enterococci in cattle manure-treated effluent were resistant to tetracycline. Enterococci were still detectable in effluent from cattle manure-treated soil columns on day 61 and 82, averaging 28 CFU 100 mL^−1^ and 32 CFU 100 mL^−1^, respectively. No enterococci were resistant to tylosin 82 days after manure application, while 25% were resistant to tetracycline.

Enterococci abundance was also determined for the soils themselves and found to be similar to the effluent results (Figure 3). Columns treated with swine manure had >10^4^ CFU g^−1^_dry_ soil on day 17 after manure application with 100% resistant to both tetracycline and tylosin. Concentrations in cattle manure-treated soil at day 17 were >10^2^ CFU g^−1^_dry_, with 100% resistant to tetracycline and 25% resistant to tylosin. Enterococci concentrations in swine manure-treated soils were considerably lower by day 89 after treatment, with an average of 5 CFU g^−1^_dry_ for swine manure and 11 CFU g^−1^_dry_ for cattle manure columns. There were no resistant enterococci detected in soil from swine manure columns at day 89 and only 9% resistant to tylosin for cattle manure-treated columns.

### 3.2. ARGs Detection Using Microfluidic qPCR

#### 3.2.1. ARGs in Swine and Cattle Source Manure Samples

**Figure 4:**
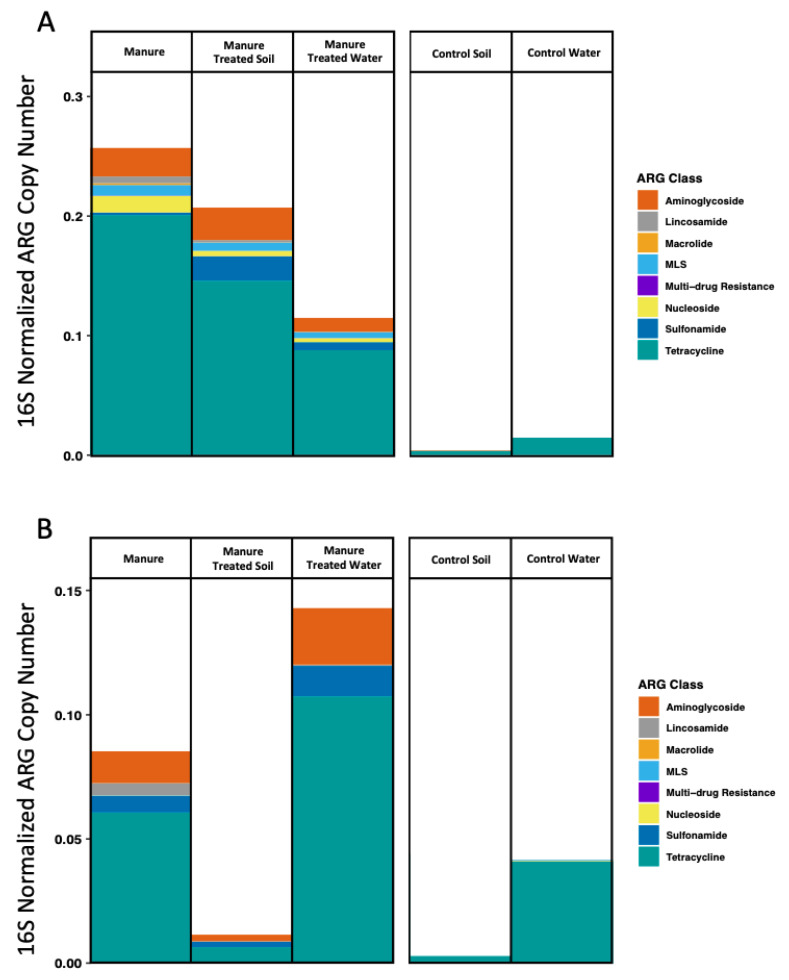
Average ARG copy numbers for source manures, effluent waters and soils for (**A**) swine manure- and (**B**) cattle manure-associated samples. Resistance genes (ARG) were clustered by class and normalized by 16S rRNA abundance.

Using microfluidic (MF) qPCR, the majority of the 36 ARGs tested were present in both manure types, with 33 detected in swine manure and 33 in cattle manure (Table 1). Thirty of the ARGs were common to both manures, with 3 ARGs unique to each manure type. ARGs that were unique among manures include *ant(6)-ia*, *erm(C)*, and *lnuC* in swine manure and *ermX*, *tetA*, *tetG* in cattle manure (Table 1). The majority of ARGs detected in source manures were associated with resistance to tetracycline and aminoglycoside antibiotics, comprising 87% and 86% of the total abundance of ARGs in swine and cattle manure respectively (Figure 4). Distinctions were seen in the remainder of the ARGs, however, with swine manure carrying mostly nucleoside (5%) and MLS (macrolide, lincosamide and streptogramin) (4%) resistance genes and cattle manure carrying mostly sulfonamide (8%) and lincosamide (5%) resistance genes. The median abundance of ARGs was significantly different between swine and cattle source manure (Mann–Whitney U test, *p* = 5.34 × 10^−5^) (Supplementary Table S8). The total abundance of ARGs detected in swine manure was approximately three times greater compared to cattle manure (Figure 4). The total abundance of ARGs associated with resistance to each class of antibiotic, with the exception of sulfonamides, was also greater in swine manure compared to cattle manure.

#### 3.2.2. Overview of ARGs in Untreated Soil and Water Samples

The median abundance of ARGs targeted in this study was not significantly different between soil and effluent water samples from control columns (Mann–Whitney U test, *p* = 0.968) (Supplementary Table S9). A total of 11 ARGs were detected in swine soil and water control samples and 10 ARGs were detected in cattle soil and water control samples, the majority of which were tetracyclines and sulfonamides in both cases (Table 1). Several genes, including *ant(6)-ib*, *sul1*, *sul2*, *tet(33)*, *tet44*, *tetG*, *tetM*, *tetO*, and *tetX*, were present in the day 0 control soil samples, despite not having manure application for at least 1 year prior to these experiments. The majority of these genes were found to decrease over the course of the experiment (see next section), although the genes *tetG* and *tet(33)* were still detectable on day 145 in swine control soil samples.

#### 3.2.3. Dynamics of ARG Abundance over Time

**Figure 5:**
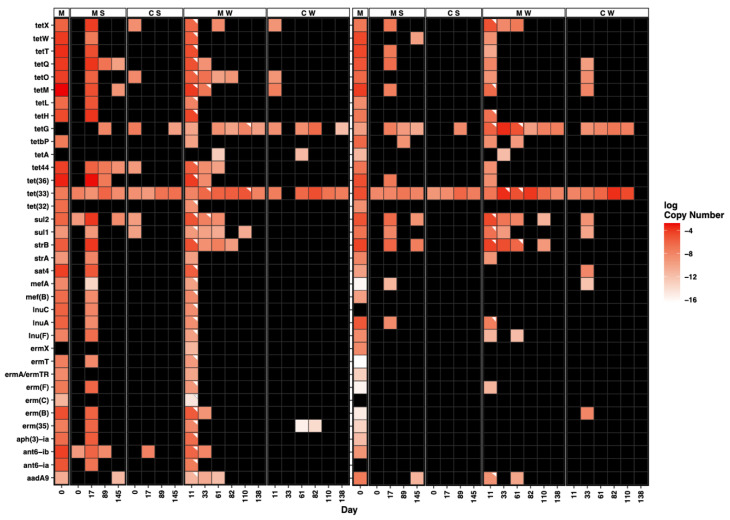
Log of mean 16S-rRNA normalized copy numbers of each ARG over time in swine or cattle manure, manure-applied (or no-manure controls) soil and effluent water samples. M: manure, MS: manure-treatment: soil, CS: Control: no-manure soil, MW: manure-treatment: drainage water, CW: control: no-manure treatment drainage water. White triangles indicate significantly increased mean abundance relative to control of the same matrix (soil or water). Black columns indicate levels were below the detection limit.

The majority of ARGs detected in swine manure were also detected in effluent water from swine manure-treated columns on day 11 at significantly increased abundances compared to background (Mann–Whitney U test, α < 0.05) (Figure 5, Supplementary Table S9). These were primarily tetracycline, aminoglycoside, MLS, lincosamide, and sulfonamide resistance genes. Some ARGs were more persistent and were detected in the majority of swine manure-treated soil samples and beyond day 11 in the manure-treated column effluent water. For example, *tetQ*, *tet(36)*, *tet44*, *tetM*, *sul2* and *ant(6)-ib* were detected on most days in swine manure-treated soil including day 145, but dropped below detection limits in swine manure-treated column effluent water within the first half of the study, indicating that they were retained in the soil more readily than other ARGs (Figure 5). Conversely, other ARGs such as *tetO*, *strB* and *sul1* were detected only on day 17 in swine manure-treated soils but persisted in swine manure-treated effluent water into the second half of the study before eventually dropping below detection limits, indicating that ARGs that are not detectable in soil may still be concentrated to detectable levels by rainfall.

Conversely, only a few of the ARGs detected in cattle manure were found at significantly increased abundances in cattle manure-treated columns compared to controls (Figure 5). The majority of these ARGs were observed on day 11 in cattle manure-treated column effluent water and were predominantly tetracycline, aminoglycoside, and lincosamide resistance genes. Only two genes, *sul2* and *strB*, continued to be enriched in cattle manure-treated soil through day 145 and both genes were also enriched in cattle manure-treated column effluent water for a substantial period of time.

#### 3.2.4. Comparison of Swine and Cattle Manure-Treated Soils and Drainage Water

**Figure 6:**
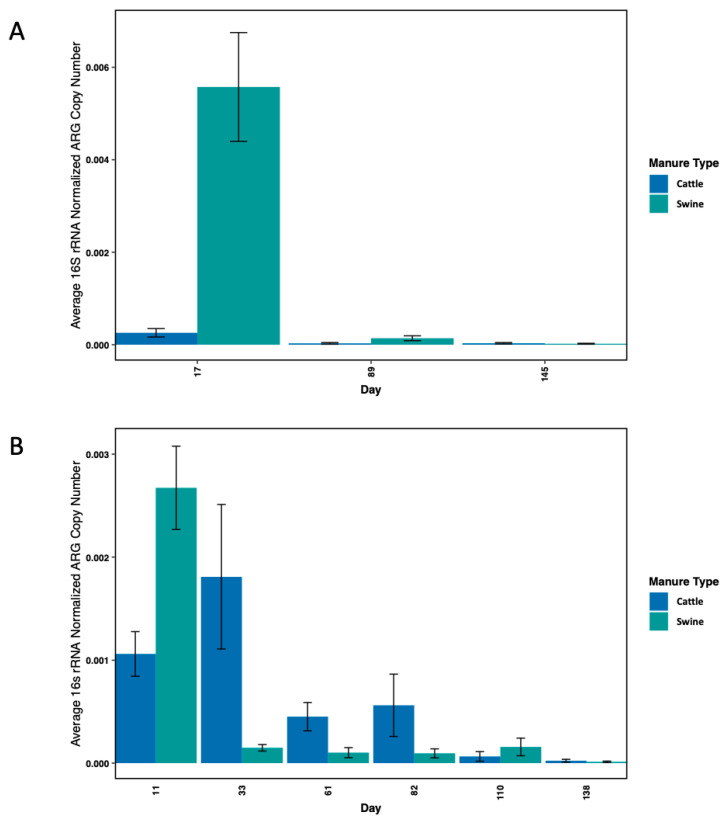
Average ARG abundances (normalized to 16s rRNA abundance) in swine and cattle manure-treated (**A**) soils and (**B**) drainage waters over time.

The median abundance of ARGs was significantly different between swine and cattle manure-treated soils and drainage waters (Mann–Whitney U test, *p* = 1.80 × 10^−11^) (Supplementary Table S9). The total abundance of ARGs in swine manure-treated soil was much greater than in cattle manure-treated soil and remained greater in soil despite a significant decrease in abundance after day 17 (Figure 6). However, the mean abundance of ARGs in cattle manure-treated column drainage water was greater compared to swine manure-treated column water, particularly after day 11. The total abundance of ARGs in swine manure-treated column water over time was characterized by a drastic reduction after day 11 followed by a more consistent abundance through the remaining days, whereas the abundance in cattle manure-treated column water spiked between days 11 and 33 followed by a gradual attenuation (Figure 6). The main drivers behind the increased ARG abundance in water leaching from cattle manure-treated columns were *tetG*, *tet(33)*, *strB*, *sul2*, and *aadA9* (Figure 5).

ARGs that were detected through the last sampling periods for each matrix, days 145 for soil and 138 for water samples, are notable because they persisted through the entire simulated growing season. The number of ARGs present on day 145 in swine and cattle manure-treated soils was the same, and most of those ARGs were shared including *aadA9*, *sul2*, *tet(33)*, and *tetG* (Figure 4). The latter two ARGs, *tet(33)* and *tetG*, also persisted through day 138 in both swine and cattle manure-treated water samples. Genes *tet(33)* and *tetG* were ubiquitous among all swine and cattle sample types including controls, but were generally more abundant in manure-treated water than controls. The *sul2* gene was unique in that it was present on most days in both swine and cattle manure-treated samples, including day 145, and persisted a substantial period of time in both swine and cattle manure-treated water samples before dropping below detection limits (undetectable on day 82 and 138 for swine and cattle, respectively).

Some ARGs showed different patterns in abundance over time between swine and cattle manure-treated soil and drainage water. For example, *tet44*, *tetQ* and *tetM* persisted through day 145 in swine manure-treated soil and were also detected after day 11 in drainage water from swine manure-treated soils, but were not detected past day 11 or 17 in soils or waters from cattle manure-treatments. The *sul1* gene was present only on day 17 in both swine and cattle manure-treated soils, but it persisted much longer in swine manure-treated water (day 110) than in cattle manure-treated water (day 33). Other ARGs also persisted much longer in drainage water from swine manure-treated soils including *sul1*, *tetO*, *tet(36)*, and *tet44*. The abundance of these ARGs was much greater in swine manure, and it is therefore likely that the difference observed relates to limit of detection for the MF-qPCR technology. Conversely, other ARGs persisted longer in drainage water from cattle manure-treated soils, including *tetbP* and *lnu(F)*, yet the abundance of these ARGs was similar between manure types.

#### 3.2.5. Detection of Mobile Genetic Elements

**Figure 7:**
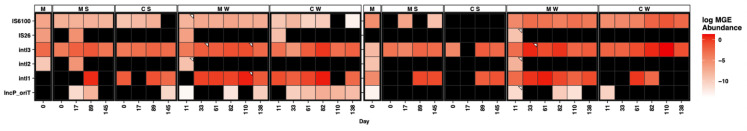
Log of mean 16S-rRNA normalized copy numbers of each MGE over time in swine or cattle manure, manure-applied soil and water and control soil and water samples. M: manure, MS: manure-treated soils, CS: control (no-manure) soil, MW: drainage water from manure-treated soil, CW: drainage water from control soils. White triangles indicate means with significantly increased abundance relative to control of the same matrix (soil or water). Black columns indicate levels were below detection limits.

Mobile Genetic Elements (MGEs) play a recognized role in the dissemination of known ARGs, and the potential emergence of novel ARGs from environmental or commensal bacteria, as they can facilitate the capture and movement of ARGs [34]. In this study, we included six MGE targets in our MF-qPCR array that have previously been identified as important in the movement of ARGs within manure and receiving environments. These targets included the three classes of mobile integrons (Int1, Int2, Int3), two related IS*6*-family elements (IS*26*, IS*6100*) and the promiscuous IncP plasmid. The MGE results could be grouped into three categories, and the patterns were similar for both manure types (Figure 7). The first group (IS*6100* and IntI3) were detected consistently in the effluent samples and at most time points for the soil samples of both manured and control soil columns. Both of these MGEs were found in higher abundance in the cattle-manure-treated soil columns compared to the swine-manure-treated. The second group (IS*26* and Int2) were clearly manure-associated and were only detected on day 11 effluent or day 17 soil in manure-treated samples. The third pattern, exhibited only by IntI1, appears to have been influenced by longitudinal factors, as levels were low or undetected in manure and increased in both the manured and control soils and effluents after the initial rain event. Finally, the IncP plasmid target was detected intermittently, but always at very low abundance.

## 4. Discussion

In this study, we employed intact soil columns with simulated rain events to evaluate the impact of swine and cattle manure on the microbial community and resistome of soil and drainage water over a field season. The study used soil from fields with a history of manure addition in order to evaluate the ongoing impact of manure application, as an extension on our earlier work examining the shifts in the microbial community when manure is initially applied to a soil environment [31]. In this study, soil columns were extracted from two separate fields that had previous manure applications of cattle or swine manure. However, the bacterial community structure of the soils on day 0 (before manure application) was similar and clearly distinct from either of the manures (Supplementary Figure S2C). This is also in agreement with our previous study indicating that manure application has a temporally limited impact on the soil microbial community structure [31]. Notably, although both manures were applied to the surface (with mixing only in the top 15 cm for swine in order to maintain pore structure in the soil columns) the impact of the manure on both the soil and effluent from the columns was clearly distinct. For the swine columns, elevated *Firmicutes* after manure application suggests that the treated soil columns continued to be significantly impacted in both soil and effluent samples throughout the experiment. Conversely, for the cattle manure-treated columns there were limited taxonomic changes beyond the first sampling date (day 11 for effluent and day 17 for soil).

Using a high-throughput microfluidic PCR approach, our study identified unique resistomes associated with each manure type and in the columns treated with each manure. The majority of the ARGs detected in manure samples were not present in either swine or cattle control soil or water samples throughout the study, indicating that the ARGs detected in manure-treated soils were derived from manures either directly (Table 1) or as a result of increased nutrient availability to the indigenous microbial communities in the soil through manure application. The swine manure resistome was primarily defined by a greater total abundance of ARGs, including those associated with resistance to every class of antibiotic represented in our study except sulfonamides. Thus, cattle manure was defined by a lower total abundance of ARGs but a higher total abundance of ARGs associated with resistance to sulfonamide antibiotics. Despite the differences observed between manures, tetracycline resistance genes comprised the overwhelming majority of ARGs detected in both swine (78%) and cattle (71%) manure. Tetracycline antibiotics are the most frequently used antibiotic in both swine and cattle in the US and the most abundant antibiotic residue detected in manure-treated soils [35,36,37], consistent with our results. In our data, the abundance of sulfonamide resistance genes *sul1* and *sul2* was 10.5 and 2.7× greater in cattle manure compared to swine manure, respectively, though specific antibiotic use regimens were not documented in this study and would be expected to influence these results. Broader management strategies within the swine and cattle industries may also influence the manure resistome in various ways, including the unique antibiotic regimens used for swine and cattle at various stages of development [38]. For example, swine are exposed to higher doses at earlier developmental stages that are tapered off towards maturity [39,40]; opposite to the typical regimen used for cattle [38]. Swine and cattle manures also have different physical and chemical properties that dictate how each manure is applied to the soil [41], and consequently how readily ARGs and ARBs associated with each manure type may be transferred from the soil and into the surrounding environment. For example, cattle manure is often stored in solid form and may be broadcast over the top of fields. Swine manure is more liquid and is commonly injected into the soil in the Midwest to help prevent volatile nitrogen losses associated with broadcasting and to decrease the runoff potential of various constituents in manure including residual antibiotics [42].

The difference in total ARG abundance observed between manure types was also observed in manure-treated soils and is consistent with observations from previous studies comparing the abundances of ARGs in raw swine and cattle manure and in manure-treated soils [18,43,44]. We generally observed an initial increase in abundance of ARGs in soil and water samples following manure application that gradually attenuated over time. However, swine and cattle manure-treated samples were each defined by unique patterns of ARG mobility through the soil and into the water as well as unique enrichments of ARGs over the course of the study. We found that in terms of total ARG abundance, swine manure impacts both the soil and water to a greater extent early in the experiment, but cattle manure impacts the water to a greater extent from day 33 forward and its impact persists for a longer period of time. For example, *sul2* and *strB* were detected on most days in cattle manure-treated soil samples, including day 145, and persisted through day 110 in cattle manure-treated water samples before dropping below detection limits, indicating that bacteria harboring these ARGs persist in the soil while posing an elevated risk for transfer into water. In addition, some ARGs (*tetX*, *sul1*) that were not detected in soils beyond day 17 were identified in water samples through the first half of the study, indicating that organisms carrying these genes may also be concentrated to detectable levels by rainfall. Despite the greater abundance of every ARG except the sulfonamide resistance genes in swine manure, cattle manure treatment had a more significant impact on the total abundance of the tetracycline, aminoglycoside, sulfonamide, and lincosamide resistance genes in water samples in the later time points. Conversely, there was consistently higher ARGs in swine manure-treated soils compared to cattle manure-treated soils between days 17 and 89. This difference was driven by several highly abundant ARGs (*tetQ*, *tet(36)*, *tet44*, *tetM*, *sul2* and *ant6-ib*) that persisted in the swine manure-treated soil through the entire study. As tetracycline is known to absorb to soil particles [45], it is likely that the bacteria harboring these ARGs are influenced by the persistence of tetracycline after manure application.

The observed differences in movement of ARGs through our model system may also partly be attributed to different moisture levels of the manures. The swine manure applied to these columns was mostly liquid, and the cattle manure was a drier solid. Although our study could not associate ARGs with specific taxa, the swine manure was distinct from the original soils and cattle manure, including a predominance of *Enterobacterales* and *Aeromonadales* (Supplementary Figure S2B), which are more commonly associated with water than soil and are known to harbor a variety of ARGs, which may account for the high ARG levels in effluent water on the first rain event after swine manure application. The high moisture content in swine manure may also allow for a more active and abundant microbial community which is reflected by the greater total abundance of ARGs compared to cattle manure. Finally, the fluid content of swine manure may also enhance the speed at which the bacterial communities in manure can move through the soil, again demonstrated by the large abundance of ARGs at early time points in swine manure-treated samples that rapidly declines following rainfall. On the other hand, the microbial community of the drier cattle manure may have been stimulated by the first simulated rainfall event on day 11, causing the spike in abundance of ARGs observed in manure-treated water samples between days 11 and 33.

Phylogenetic analysis of the swine soil column effluent showed a clear impact of manure addition through the duration of the trial, yet ARG abundance was primarily impacted in the early effluent samples. This suggests that our limit of detection for ARG analysis was a potential limiting factor, which is to be expected from a low input method such as the MF-qPCR approach used here. Future studies aimed at improving DNA extraction from complex, organic-rich samples would benefit this area of research, along with exploring the use of pre-amplification to increase detection for MF-qPCR. This study had reduced sensitivity for detecting resistance genes than our previous work using shotgun metagenomics [18]; however, the significant cost reductions allowed for a much larger number of samples to be analyzed and avoided the known issues of non-target sequences generated through shotgun approaches. Notably, comparison of the MF-qPCR results to counts of Enterococci supports the practical application of our method. Enterococci are a common fecal indicator bacteria (FIB) used to determine water contamination from sewage or agricultural sources. In both the swine and fecal soil columns, FIB were undetectable by half way through the trial (day 61 for swine and day 82 for cattle columns) and with the exception of *tet33* and *tetG* (discussed below), there were no significantly enriched ARGs beyond this time point. The comparability to standard FIB testing suggests that the use of MF-qPCR could be an informative method for evaluating fecal contamination and future studies should investigate its suitability for incorporation into human health risk assessments of water quality.

We identified several ARGs in manure-treated soil and drainage water that may serve as relevant targets for future monitoring efforts. The ARGs driving the spike in abundance in cattle manure-treated water between days 11 and 33 were *tetG*, *tet(33)*, *strB*, *sul2*, and *aadA9*. Most of these ARGs were also present in both swine and cattle manure-treated soils through the entire study and also persisted a substantial period of time in drainage water from manure-treated soils. Genes *tet(33)* and *tetG* were ubiquitous among all swine and cattle sample types including controls which, as noted, also had historical manure application and suggests that some ARGs can persist in the soil for extended periods.

All of these potential target ARGs have previously been associated with mobile elements, and many have been observed together within the same mobile elements. Genes *aadA9* and *tet(33)* commonly occur on multi-drug resistant conjugative plasmids, for example pTET3 that also harbors multiple copies of IS*6100* and a class 1 integron [46]. The *tet(33)* gene was also identified as unique in our previous column study because it was the only ARG observed in swine manure-treated soil and water metagenomes that increased in abundance over time [18]. The *tetG* gene is found on chromosomes and plasmids of Gram-negative bacteria and is frequently linked with *sul1* [47]. Genes *strB* and *sul2* are both found in Gram-negative bacteria and are often observed clustered together [48,49]. The *strB* gene has been found on diverse mobile elements including plasmids, integrated conjugative elements, and chromosomal genomic islands [50,51]. Additionally, *aadA9* and *sul2* have been previously identified as integron cassettes [52,53]. Notably, in our study, the mobile elements IS*6100* and IntI3 were also found to be present in both treated and control (with manure history) soils throughout the study and the class 1 integron (IntI1) increased in abundance over the course of the experiment. This suggests that these mobile elements may also be suitable targets for monitoring long-term manure impact, as has been shown previously for IntI1 in relation to anthropogenic pollution [54]. In addition, IS*26* and IntI2 were transiently observed in the manure-treated columns and may therefore be useful targets for recent manure impact.

## 5. Conclusions

In this study, soil columns were extracted from fields with a history of manure application and the impact of fresh manure was evaluated after simulated rain events over the course of a field season. In support of our first hypothesis, we found significant differences in the microbial composition and resistomes of source manures from swine and cattle that were distinguishable by unique abundances and types of ARGs. The distinctions observed between manure resistomes were also observed in the manure-treated soil and drainage water of our model system and contributed to variable patterns observed in the movement of ARGs from the manure, through the soil and into the water, supporting our second hypothesis. Future studies should focus on identifying the bacterial hosts of these ARGs and the environmental or physicochemical properties that influence their transport through agricultural systems. In addition, this work needs to be repeated using manure from diverse sources, preferably with complete antimicrobial usage information, to confirm the general applicability of our results. Finally, several ARGs were identified that were significantly increased in drainage water following manure application, some of which persisted long term in the soil and drainage water. This work demonstrates the capabilities for targeted PCR surveillance of resistance genes and mobile elements to identify agricultural impacts on the environment, and these tools can be implemented in future studies to evaluate the impact of manure management and antimicrobial use policies on resistance gene dissemination.

## Figures and Tables

**Table 1:** Detection (indicated by a circle) of antibiotic resistance genes in swine and cattle manures compared to detection in soil without manure (controls). Class and resistance mechanism information for each gene was obtained from the Comprehensive Antimicrobial Resistance Database (https://card.mcmaster.ca/).

Genes	Swine Manure	Swine Control	Cattle Manure	Cattle Control	Class	Resistance Mechanism
*tet(33)*	●	●	●	●	Tetracycline	Antibiotic efflux
*tetO*	●	●	●	●	Tetracycline	Antibiotic target protection
*tet44*	●	●	●		Tetracycline	Antibiotic target protection
*tetX*	●	●	●		Tetracycline, glycylcline	Antibiotic inactivation
*tetQ*	●		●	●	Tetracycline	Antibiotic target protection
*tetT*	●		●		Tetracycline	Antibiotic target protection
*tetW*	●		●		Tetracycline	Antibiotic target protection
*tet(36)*	●		●		Tetracycline	Antibiotic target protection
*tetbP*	●		●		Tetracycline	Antibiotic efflux
*tetH*	●		●		Tetracycline	Antibiotic efflux
*tetL*	●		●		Tetracycline	Antibiotic efflux
*tetM*	●	●	●	●	Tetracycline	Antibiotic target protection
*tet(32)*	●		●		Tetracycline	Antibiotic target protection
*mefA*	●		●	●	Tetracycline, lincosamide, phenicol, pleuromutilin, oxazolidinone, streptogramin, macrolide	Antibiotic target protection
*erm(35)*	●	●	●		Streptogramin, macrolide, lincosamide	Antibiotic target alteration
*erm(B)*	●		●	●	Streptogramin, macrolide, lincosamide	Antibiotic target alteration
*ermA*	●		●		Streptogramin, macrolide, lincosamide	Antibiotic target alteration
*erm(F)*	●		●		Streptogramin, macrolide, lincosamide	Antibiotic target alteration
*ermT*	●		●		Streptogramin, macrolide, lincosamide	Antibiotic target alteration
*mef(B)*	●		●		Macrolide	Antibiotic efflux
*ant6-ib*	●	●	●		Aminoglycoside	Antibiotic inactivation
*strB*	●		●		Aminoglycoside	Antibiotic inactivation
*aadA9*	●		●		Aminoglycoside	Antibiotic inactivation
*strA*	●		●		Aminoglycoside	Antibiotic inactivation
*aph(3)-ia*	●		●		Aminoglycoside	Antibiotic inactivation
*sul2*	●	●	●	●	Sulfonamide	Antibiotic target replacement
*sul1*	●	●	●	●	Sulfonamide	Antibiotic target replacement
*lnuA*	●		●		Lincosamide	Antibiotic inactivation
*lnu(F)*	●		●		Lincosamide	Antibiotic inactivation
*sat4*	●		●	●	Nucleoside	Antibiotic inactivation
*tetG*		●	●	●	Tetracycline	Antibiotic efflux
*tetA*		●	●		Tetracycline	Antibiotic efflux
*ermX*			●		Streptogramin, macrolide, lincosamide	Antibiotic target alteration
*erm©*	●				Streptogramin, macrolide, lincosamide	Antibiotic target alteration
*ant6-ia*	●				Aminoglycoside	Antibiotic inactivation
*lnuC*	●				Lincosamide	Antibiotic inactivation

## Data Availability

Amplicon sequence data were deposited to the NCBI SRA under Bioproject ID PRJNA820041. All metagenomes were deposited in NCBI SRA accession number PRJNA662623.

## Supplementary Materials

The following are available online at https://www.mdpi.com/article/10.3390/microorganisms11010017/s1. Figure S1: Soil column setup illustrating end caps with drainage tubes; Figure S2: Taxa distribution of (A) source manures at phylum level with swine dominated phyla on the positive y-axis and beef dominant phyla on the negative y-axis (B) Proteobacteria within source manures at the Order level and (C) top families from soil columns on day 0; Figure S3: Abundance of 5 key phyla in soil from cattle-manure treated columns over time; Table S1: Primer sequences used in this study; Table S2: Samples pooled to create interplate calibrator samples; Table S3: Standard sequences for each primer set with amplification efficiencies and limits of detection; Table S4: Number of biological and technical replicates for each sample type; total replicates in data output; Table S5: ANCOM-BC analysis of effluent water from swine treated columns on phyla level taxa abundance over time. Table S6: ANCOM-BC analysis of soil from swine treated columns on phyla level taxa abundance over time; Table S7: ANCOM-BC analysis of effluent water from beef treated columns on phyla level taxa abundance over time; Table S8: Mann-Whitney U-test p-values for significant differences in median abundance of ARGs between swine and beef manure sample types, combined control samples, and manure-treated samples; Table S9: Mann-Whitney U-test p-values for differences in median abundance of ARGs in swine or beef manure-treated samples and paired controls.
