# Supplementary material for: Impact of Swine and Cattle Manure Treatment on the Microbial Composition and Resistome of Soil and Drainage Water
Source: Microorganisms. 2022 Dec 21;11(1):17. doi: 10.3390/microorganisms11010017 (PMC9865870; doi:10.3390/microorganisms11010017)
Supplement: Supplementary file 1 [file microorganisms-11-00017-s001.zip › microorganisms-2016687-supplementary.pdf]

Supplementary Information

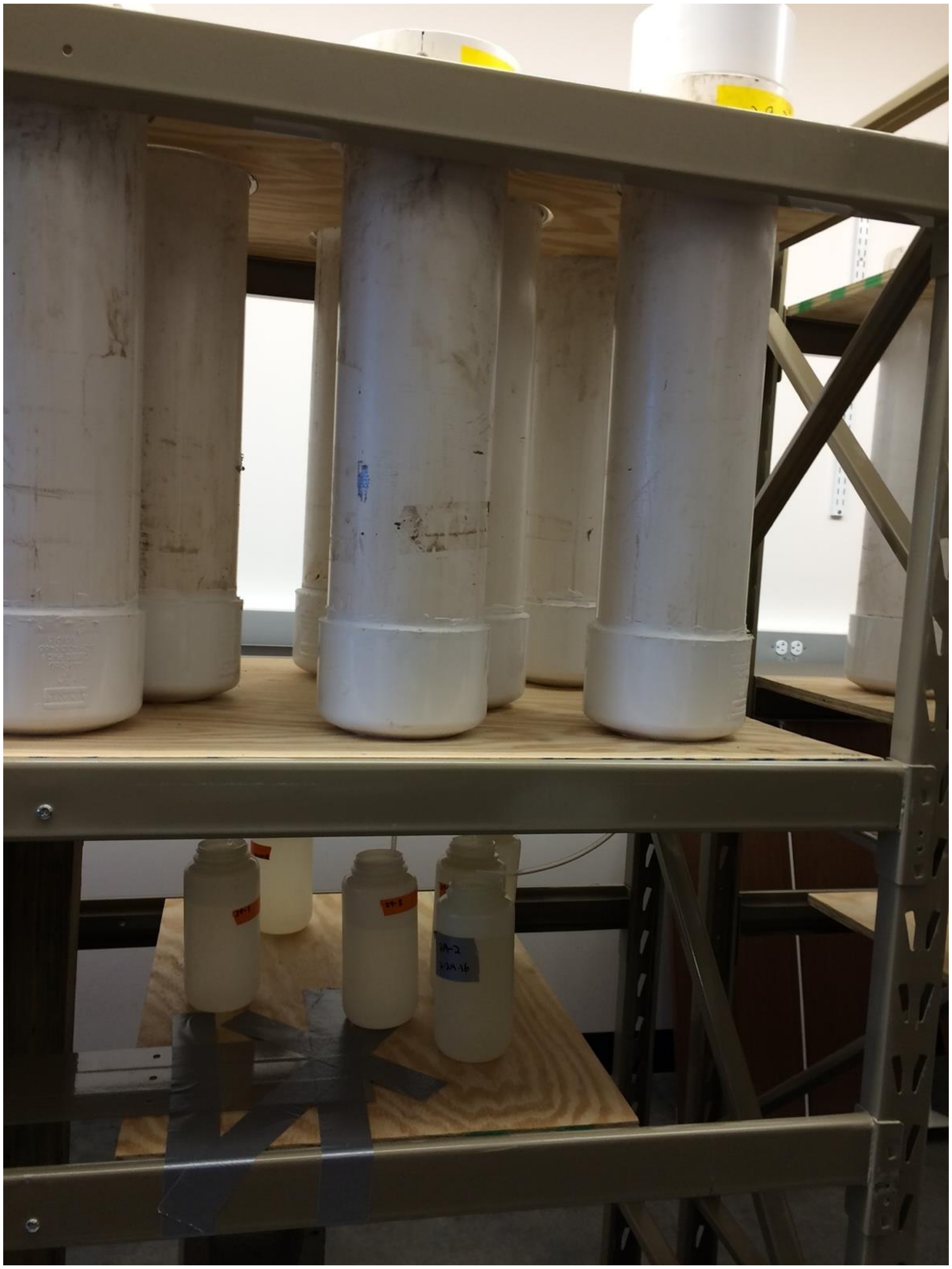

Figure S1: Soil column setup illustrating end caps with drainage tubes.

Table S1 – Primer sequences used in this study. Primers were primarily obtained from Stedtfeld et al. (FEMS Microbiol Ecol ***2018,*** *94 (9)) except for the 16s_Eub primers (López-Gutiérrez et al., 2004), 16S_1055yF (Dionisi et al. Appl Environ Microbiol. 2003 Nov;69(11):6597-604), 16s_1392_R (Ritalahti et al. Appl Environ Microbiol. 2006 Apr;72(4):2765-74) and tet36, tet33 primers that were developed in-house for our purposes.*

Name	Sequence	Tm (℃)
aadA9_F	CGCGGCAAGCCTATCTTG	57
aadA9_R	CAAATCAGCGACCGCAGACT	58
ant6-ia_F	TCGCCATGAGCTGCTGA	57
ant6-ia_R	CCTATCATACTCCGGATAGGCATA	55
ant6-ib_F	AGAACATCCGACAGCACGTTC	58
ant6-ib_R	CCAACCTTCCATGAAATCATTCGC	57
aph(3'')-ia_F	TAACAGCGATCGCGTATTTCG	55
aph(3'')-ia_R	TCCGACTCGTCCAACATCAATA	56
erm(35)_F	CCTTCAGTCAGAACCGGCAA	58
erm(35)_R	GCTGATTTGACAGTTGGTGGTG	57
erm(B)_F	GAACACTAGGGTTGTTCTTGCA	55
erm(B)_R	CTGGAACATCTGTGGTATGGC	56
erm(C)_F	TTTGAAATCGGCTCAGGAAAA	53
erm(C)_R	ATGGTCTATTTCAATGGCAGTTACG	55
erm(F)_F	TCTGATGCCCGAAATGTTCAAG	56
erm(F)_R	TGAAGGACAATTGAACCTCCCA	56
ermA/ermTR_F	ACATTTTACCAAGGAACTTGTGGAA	55
ermA/ermTR_R	GTGGCATGACATAAACCTTCATCA	56
ermT_F	GTTCACTAGCACTATTTTTAATGACAGAAGT	56
ermT_R	GAAGGGTGTCTTTTTAATACAATTAACGA	54
ermX_F	GCTCAGTGGTCCCCATGGT	60
ermX_R	ATCCCCCCGTCAACGTTT	57
IncP_oriT_F	CAGCCTCGCAGAGCAGGAT	60
IncP_oriT_R	CAGCCGGGCAGGATAGGTGAAGT	63
int1-a-marko_F	CGAAGTCGAGGCATTTCTGTC	56
int1-a-marko_R	GCCTTCCAGAAAACCGAGGA	57
intI1F165_clinical_F	CGAACGAGTGGCGGAGGGTG	63
intI1F165_clinical_R	TACCCGAGAGCTTGGCACCCA	63
intl2_F	TGCTTTTCCCACCCTTACC	55
intl2_R	GACGGCTACCCTCTGTTATCTC	56
intl3_F	CAGGTGCTGGGCATGGA	58
intl3_R	CCTGGGCAGCATCACCA	58

IS26_F	ATGGATGAAACCTACGTGAAGGTC	57
IS26_R	CGGTACTTAATCTGTCGGTGTTCA	57
IS6100_F	CGCACCGGCTTGATCAGTA	58
IS6100_R	CTGCCACGCTCAATACCGA	58
lnu(F)_F	ATACCGGTCATTTCCACTTGGC	58
lnu(F)_R	GCATCAGGCTGATGAGGTTCAA	58
lnuA_F	TGACGCTCAACACACTCAAAAA	55
lnuA_R	TTCATGCTTAAGTTCCATACGTGAA	55
lnuC_F	GGGTGTAGATGCTCTTCTTGGA	56
lnuC_R	CTTTACCCGAAAGAGTTTCTACCG	55
mef(B)_F	CCGATAGGCTTACTTGTTGCAG	56
mef(B)_R	AGTCCACTTGCGGTTTCATTG	56
mefA_F	TAATTATCGCAGCAGCTGGTTC	55
mefA_R	GTTCCCAAACGGAGTATAAGAGTG	55
sat4_F	GAATGGGCAAAGCATAAAAACTTG	54
sat4_R	CCGATTTTGAAACCACAATTATGATA	52
strA_F	CCGGTGGCATTTGAGAAAAA	54
strA_R	GTGGCTCAACCTGCGAAAAG	57
strB_F	GCTCGGTCGTGAGAACAATCT	57
strB_R	CAATTTCGGTCGCCTGGTAGT	57
sul1 NEW_F	GCCGATGAGATCAGACGTATTG	55
sul1 NEW_R	CGCATAGCGCTGGGTTTC	57
sul2_F	TCATCTGCCAAACTCGTCGTTA	56
sul2_R	GTCAAAGAACGCCGCAATGT	57
tet(32)_F	CCATTACTTCGGACAACGGTAGA	56
tet(32)_R	CAATCTCTGTGAGGGCATTTAACA	55
tet(36)_F	AGAATACTCAGCAGAGGTCAGTTCCT	59
tet(36)_R	TGGTAGGTCGATAACCCGAAAAT	56
tet44_F	CTCATGTAGATGCAGGAAAGACG	55
tet44_R	GTAACTGCTGCCTGAATTGTGA	56
tetA_F	CTCACCAGCCTGACCTCGAT	59
tetA_R	CACGTTGTTATAGAAGCCGCATAG	56
tetbP_F	TGGGCGACAGTAGGCTTAGAA	58
tetbP_R	TGACCCTACTGAAACATTAGAAATATACCT	56
tetG_F_F	TCGCGTTCCTGCTTGCC	59
tetG_F_R	CCGCGAGCGACAAACCA	59
tetH_F	TTTGGGTCATCTTACCAGCATTAA	55
tetH_R	TTGCGCATTATCATCGACAGA	55
tetL_F	ATGGTTGTAGTTGCGCGCTATAT	57
tetL_R	ATCGCTGGACCGACTCCTT	58

tetM_F	GGAGCGATTACAGAATTAGGAAGC	56
tetM_R	TCCATATGTCCTGGCGTGTC	57
tetO_F	CAACATTAACGGAAAGTTTATTGTATACCA	54
tetO_R	TTGACGCTCCAAATTCATTGTATC	54
tetQ_F	CGCCTCAGAAGTAAGTTCATACACTAAG	57
tetQ_R	TCGTTCATGCGGATATTATCAGAAT	54
tetT_F	CCATATAGAGGTTCCACCAAATCC	55
tetT_R	TGACCCTATTGGTAGTGGTTCTATTG	56
tetW_F	ATGAACATTCCCACCGTTATCTTT	55
tetW_R	ATATCGGCGGAGAGCTTATCC	56
tetX_F	AAATTTGTTACCGACACGGAAGTT	55
tetX_R	CATAGCTGAAAAAATCCAGGACAGTT	56
tet33_241_388_F	TGCTTGTTTCCCTGGCCG	54
tet33_241_388_R	GCGTGATGTCGGCGATCA	55
16S_Eub_338_F	ACTCCTACGGGAGGCAGCAG	58
16S_Eub_518_R	ATTACCGCGGCTGCTGG	54
16S_1055yF	ATGGTTGTCGTCAGCT	45
16S_1392R	ACGGGCGGTGTGTAC	47

Table S2 – Samples pooled to create interplate calibrator samples. The concentration of this calibrator sample was 11.04 ng/ul.

Pooled Sample ID	Manure Type	Treatment	Matrix	Day
A224Pman1	Swine	Manure	Manure	0
A792-794BMaS17C01	Beef	Manure	Soil	17
A797-799BMaS17C02	Beef	Manure	Soil	17
A193-195BCoR33C01	Beef	Control	Water	33
A246-248BMaR33C09	Beef	Manure	Water	33
A300-302PCoR61C10	Swine	Control	Water	61
A522-524BMaR110C09	Beef	Manure	Water	110
A598-600PCoR138C10	Swine	Control	Water	138
A568-570BMaR138C03	Beef	Manure	Water	138
A902-904PCoS145C04	Swine	Control	Soil	145

Table S3 – Standard sequences for each primer set with amplification efficiencies and limits of detection

Primer Name	Standard Length (bp)	Limit of Detection (copies/gram of soil of mL of water)	R2	Efficiency (%)	Standard Sequence
16S_Eub_338	257	24	0.9981	126	ACTGTCAAGCTTAGCCAATACTGGTTGAGGACTCCTACGGGAGGCAGCAGTAGGGAATCTTCCACAATGGACGAAAGTCTGATGGAGCAACGCCGCGTGAGTGAAGAAGGGTTTCGGCTCGTAAAACTCTGTTGTTAAAGAAGAACATATCTGAGAGTAACTGTTCAGGTATTGACGGTATTTAACCAGAAAGCCACGGCTAACTACGTGCCAGCAGCCGCGGTAATTCCTTCTTCAACAATCAGAAGCTTACTGTC
aadA9	251	24	0.9942	132	ACTGTCAAGCTTATGTGGCCGCCGAATGGGTTTTAGAGCGCTTGCCAGCTGAGCATAAGCCAATACTGGTTGAGGCGCGGCAAGCCTATCTTGGGCTTTGCAAGGATAGTCTTGCTTTGCGTGCAGATGAGACTTCGGCGTTCATTGGCTATGCAAAGTCTGCGGTCGCTGATTTGCTCGAAAAGCGAAAATCTCAAACTTCGCATATTTGCGATGGCGCCAAGAACGTCTAACGTGATAAGCTTACTGTC
ant6-ia	313	20	0.9925	109	ACTGTCAAGCTTAGCCAATACTGGTTGAGGTCGCCATGAGCTGCTGAGAATGATATCATGGAAGGTCGGCATCGAAACAGGCTTTAAATTAAGTGTAGGCAAGAACTATAAGTTTATTGAAAGGTATATATCCGAGGATTTGTGGGAGAAACTTTTGTCCACCTACCGGATGGATTCCTATGAAAACATATGGGAAGCATTATTTCTATGCCATCAATTGTTCAGGGCGGTATCCGGTGAGGTGGCGGAAAGGCTTCATTATGCCTATCCGGAGTATGATAGGTCCTTCTTCAACAATCAGAAGCTTACTGTC
ant6-ib	251	24	0.9975	110	ACTGTCAAGCTTTACCAGTACCTACAGATGAAGATTATTATATAGAACATCCGACAGCACGTTCTTTTGATGATTGCTGTAATGAATTTTGGAATACTGTAACATATGTAGTGAAAGGATTATGTCGAAAGGAAATTTTATTTGCAATCGACCATTTAAATAATATTGTGCGTATGGAATTACTGCGAATGATTTCATGGAAGGTTGGAATAGAGCAAGGTTATAGTTTTAGTCTAGGAAAGCTTACTGTC
aph(3'')-ia	301	20	0.9971	112	ACTGTCAAGCTTAGCCAATACTGGTTGAGGTAACAGCGATCGCGTATTTCGTCTCGCTCAGGCGCAATCACGAATGAATAACGGTTTGGTTGATGCGAGTGATTTTGATGACGAGCGTAATGGCTGGCCTGTTGAACAAGTCTGGAAAGAAATGCATAAACTTTTGCCATTCTCACCGGATTCAGTCGTCACTCATGGTGATTTCTCACTTGATAACCTTATTTTTGACGAGGGGAAATTAATAGGTTGTATTGATGTTGGACGAGTCGGATCCTTCTTCAACAATCAGAAGCTTACTGTC

erm(35)	251	24	0.9858	136	ACTGTCAAGCTTATGTTTGAAAATGTCGAATATTTTCTATGCGGTTCAATTATCCTTCAGTCAGAACCGGCAAAAAAATTGTTTTCAAGTAAGGTTTATAACCCATTGACAGTACTTTATCATACCTATTATGATTTGAAATTCCTGTATGAGATAAATCCTGAAAGTTTTTTGCCACCACCAACTGTCAAATCAGCACTTTTGAGAATTGAAAGAAAACAGATTTCATTAGATATTGGAAGCTTACTGTC
erm(B)	251	24	0.9970	118	ACTGTCAAGCTTTTGACCCATTTTGAAACAAAGTACGTATATAGCTTCCAATATTTATCTGGAACATCTGTGGTATGGCGGGTAAGTTTTATTAAGACACTGTTTACTTTTGGTTTAGGATGAAAGCATTCCGCTGGCAGCTTAAGCAATTGCTGAATCGAGACTTGAGTGTGCAAGAACAACCCTAGTGTTCGGTGAATATCCAAGGTACGCTTGTAGAATCCTTCTTCAACAATCAGAAGCTTACTGTC
erm(C)	251	24	0.9938	152	ACTGTCAAGCTTATAATGACAAATATAAGATTAAATGAACATGATAATATCTTTGAAATCGGCTCAGGAAAAGGGCATTTTACCCTTGAATTAGTACAGAGGTGTAATTTCGTAACTGCCATTGAAATAGACCATAAATTATGCAAAACTACAGAAAATAAACTTGTTGATCACGATAATTTCCAAGTTTTAAACAAGGATATATTGCAGTTTAAATTTCCTAAAAACCAATCCTATAAAAGCTTACTGTC
erm(F)	251	24	0.9987	116	ACTGTCAAGCTTCTTTGGTTGAACATTTACGAAAATTATTTTCTGATGCCCGAAATGTTCAAGTTGTCGGTTGTGATTTTAGGAATTTTGCAGTTCCGAAATTTCCTTTCAAAGTGGTGTCAAATATTCCTTATGGCATTACTTCCGATATTTTCAAAATCCTGATGTTTGAGAGTCTTGAAAATTTTCTGGGAGGTTCAATTGTCCTTCAGTTAGAACCTACACAAAAGTTATTTTCGAAGCTTACTGTC
ermA/ermTR	251	24	0.9914	133	ACTGTCAAGCTTGCATGTAAAGGAAATATTAAAATATACGAATATCAATAAACAAGATAAAATAATAGAAATTGGGTCAGGAAAAGGACATTTTACCAAGGAACTTGTGGAAATGAGTCAACGGGTGAATGCTATAGAGATTGATGAAGGTTTATGTCATGCCACGAAAAAAGCAGTTGAACCTTTTCAGAATATAAAAGTTATTCATGAGGATATTTTGAAGTTTAGCTTTCCTAAAAAAGCTTACTGTC
ermT	251	21	0.9977	124	ACTGTCAAGCTTCCATTTCATAACAAAATTTTCATATTGTTTTCTATCTTTGAGTGATATTTTTGAAGGGTGTCTTTTTAATACAATTAACGAGCTATTAACTCTAGGTTTTGGATGAAAGTATTCTCTAGGGATTTTACTTAATATGGATATATCAACTTCTGTCATTAAAAATAGTGCTAGTGAACGATTTGTATTTAGCAACCTTTTAGCAAATCCATATTCCACTATTAAATAACAAGCTTACTGTC

ermX	251	24	0.9985	116	ACTGTCAAGCTTACCATCGCCTGAAAGGCTTTGCGCTGTTCTATCGGAATCTTCGGGTCACCCACCCGGCGGATCACTAAGATCCCCCCGTCAACGTTTGGCTGTGGCCGGAAAGCAGTCCTTGGTACCCGAGAACCCAGGTGAAATGTGAACCATGGGGACCACTGAGCCGTCATCATCGTGCTTGCGCCTACCCCGGCCCGGCGGCGAGCGACTTCCCACTGCATGAGGAGTACAGCAAGCTTACTGTC
lnu(F)	251	24	0.9963	140	ACTGTCAAGCTTCGATTTAAAAGATTGGCCCCAAAGAGCATCAGGCTGATGAGGTTCAACACAAGCCCTTCCACCAGCGGCGCGCCTTCACGTATCGGGGGATCGCCCACGAGAGCGCTTGCATACCTTGACAACTCTCCTGATCGGTCCAACAAAACAGCCGCCTCAAGCGAGGGAAACCACCCATAGCCTTGCCAAGTGGAAATGACCGGTATGTCCGATTTTCGCATGAAATGGAAAAGCTTACTGTC
lnuA	251	24	0.9960	114	ACTGTCAAGCTTTTGCTTGGGTAATTGATCCATCATCATTTAGATTTATAGGATGAATATCTAAATACCCATATTCTTCATGCTTAAGTTCCATACGTGAAGGCATCCAATCAACTTCTATTTTATATCCGATATCTTCTAATTTTTGTATAACTTTTTGAGTGTGTTGAGCGTCAAAATCTATATCTATATCTCTGTGTTCTCTTTGTTGTTTTCCAGTTAATACATCTACCCCCCAGAAGCTTACTGTC
lnuC	326	19	0.9895	117	ACTGTCAAGCTTAGCCAATACTGGTTGAGGGGGTGTAGATGCTCTTCTTGGATATCAGTCAAGAGCCCATAATGATATTGACATTTTTGTAGAAAAGAACGATTATCAGAACTTTATAGAAATAATGAAAGCTAATGGCTTTTATGAGATTAAGATGGAATATACAACATTGAACCATACTGTATGGGAAGATTTGAAAAACAGAATTATTGATTTGCATTGTTTTGAATATACGGACGAAGGTGAAATTCTTTATGATGGGGATTGTTTTCCGGTAGAAACTCTTTCGGGTAAAGTCCTTCTTCAACAATCAGAAGCTTACTGTC
mef(B)	251	24	0.9974	108	ACTGTCAAGCTTGTTTTCCCTTTTGATGACCGCCATGACTCTTTCTATGCCGATAGGCTTACTTGTTGCAGGTCCGGTTGTTGAGGTTATAGGTGTTAATACATGGTTTTTCTGGTCTGGTGTTGCGTTGATAGTAAACGCTGTTCTCTGCCGCATTCTGACACGACGCTATGACAAAGTAACAATGAAACCGCAAGTGGACTGAAAAAAGGACCGGGTTGATGATAATTTGTAGTGGTAAGCTTACTGTC
mefA	315	19	0.9971	140	ACTGTCAAGCTTAGCCAATACTGGTTGAGGTAATTATCGCAGCAGCTGGTTCGGTGCTTACTATTGTTGCATTCTATATGGAGCTACCTGTCTGGATGGTTATGATAGTATTGTTTATCCGTAGCATTGGAACAGCTTTTCACACCCCGGCTCTCAATGCGGTTACGCCACTTTTAGTACCAGAAGAACAGCTTACGAAATGTGCAGGCTATAGTCAGTCTTTGCAGTCTATAAGCTATATTGTTAGTCCGGCAGTTGCAGCACTCTTATACTCCGTTTGGGAACTCCTTCTTCAACAATCAGAAGCTTACTGTC

sat4	251	24	0.9927	113	ACTGTCAAGCTTGTCGAGAGTAGTGCGTCCTGCGGAGCTTCATTCCCGGTCAGCGCGCTTATCAATATATCTATAGAATGGGCAAAGCATAAAAACTTGCATGGACTAATGCTTGAAACCCAGGACAATAACCTTATAGCTTGTAAATTCTATCATAATTGTGGTTTCAAAATCGGCTCCGTCGATACTATGTTATACGCCAACTTTGAAAACAACTTTGAAAAAGCTGTTTTCTGGTAAAGCTTACTGTC
strA	251	24	0.9985	133	ACTGTCAAGCTTTTCGAATATATAAATGGCTTTTATAATCCACGCCGAAGACATTCAACACTCGGCTGGAAATCGCCGGTGGCATTTGAGAAAAAAGCCGCTTAAAATGAGAGATAGACCGGAACACAACCGGTGCAAGTCCATAGCAACTCGTTTCTTTTCGCAGGTTGAGCCACCTCCGCGCTTCATCAGAAAACTGAAGGAACCTCCATTGAATCGAACTAATATTTTTTTGGTGAAAGCTTACTGTC
strB	251	24	0.9981	109	ACTGTCAAGCTTGGAGAAGGGCAGAAGGCAGGGGTTCCTCAGATGCGGCATACAGCTTCGCCATTAGTTCCGCTGCAATTTCGGTCGCCTGGTAGTCGCCGTGCTCGGCAACGATGTGAGAGAGCATTCGCTCCCCGGCATATTCGAGCAACATCAGATTGTTCTCACGACCGAGCAACCGGACTGCTCCCCTCCCATTGCGCCATACCAGATAGTCGGCCCCGCGCAGTTCATCAGCAAAGCTTACTGTC
sul1	251	24	0.9997	128	ACTGTCAAGCTTGGATCAGACGTCGTGGATGTCGGACCGGCCGCCAGCCATCCGGACGCGAGGCCTGTATCGCCGGCCGATGAGATCAGACGTATTGCGCCGCTCTTAGACGCCCTGTCCGATCAGATGCACCGTGTTTCAATCGACAGCTTCCAACCGGAAACCCAGCGCTATGCGCTCAAGCGCGGCGTGGGCTACCTGAACGATATCCAAGGATTTCCTGACCCTGCGCTCTATCCAAGCTTACTGTC
sul2	251	24	0.9984	112	ACTGTCAAGCTTGGCCTATCTCAATGATATTCGCGGTTTTCCAGACGCTGCGTTCTATCCGCAATTGGCGAAATCATCTGCCAAACTCGTCGTTATGCATTCGGTGCAAGACGGGCAGGCAGATCGGCGCGAGGCACCCGCTGGCGACATCATGGATCACATTGCGGCGTTCTTTGACGCGCGCATCGCGGCGCTGACGGGTGCCGGTATCAAACGCAACCGCCTTGTCCTTGATCCCGAAGCTTACTGTC
tet(32)	251	24	0.9920	123	ACTGTCAAGCTTACTTTTCCCAAAAAAGATAATATAATCTCATGTGTAACAGTATCAATGTCAAAATGCAAAAGAGGGTCTGTATCAGCAATCTCTGTGAGGGCATTTAACAGGGCTTCCCTTTGCTCCGGCTTTTGCGGCTCTACCGTTGTCCGAAGTAATGGCATGGGATTATCAATCCGTGTTTTGTGAGGCAGGAGTTTTTCATTTCCCAGAATGTCGTTCAGTTTCAAAGTATCAAGCTTACTGTC

tet(33)	251	24	0.9 901	96	ACTGTCAAGCTTGCACTGAGTAAACCGAAACGCTTGGCGCGCTGGTGGGGTGGCGTGATGTCGGCGATCACGGTGGCGGTGACCGCATTGGTCGCTCCGGTTATCCCAGCCACTGCGCGGGCGATATAGAACACCGACAGAGCGGACGTCGTGGCGAGCACGAGATAGTCGACGGTCGCACCGGCCAGGGAAACAAGCAGCACCCGGCGGCGGCCGAATCGGTCCGACAGTGTTCCCAGAAGCTTACTGTC
tet(36)	251	24	0.9 976	107	ACTGTCAAGCTTGGCGAAATATCTACTATTACTTCAGATGGTGATTGGACTACTATTTTGGGTAATATTCCTTTAGATACTAGTAAAGAATACTCAGCAGAGGTCAGTTCCTACACACAAGGCTTGGGCGTTTTTGTTACTCGATTTTCGGGTTATCGACCTACCAACAAAAAGGTAAGCAGAAGTGTAGAACTGAATGAAAAAGATAAGCTGATGTATATGTTTGAGAAGGAAAGTATAAGCTTACTGTC
tet44	251	24	0.9 947	100	ACTGTCAAGCTTTACAATATTGATTTTGTAGTCATTCCAATTAAAAGAAGTAACTGCTGCCTGAATTGTGATTCCACGCTGACGTTCTAAAAACATAGTATCTGTCCTTGTTGTTCCCTTATCTACACTGCCTAATTCTAAAATCGCTCCACTTGTATAAAGCAGACTTTCCGTTAAAGTCGTCTTTCCTGCATCTACATGAGCAAGAATACCAATGTTGATTATTTTCATGGAATTTCAAGCTTACTGTC
tetA	251	24	0.9 963	113	ACTGTCAAGCTTCCGCTCCAAAGCCCGCGACGCAGCGCCGGCAGGCAGAGCAAGTAGAGGGCAGCGCCTGCAATCCATGCCCACCCGTTCCACGTTGTTATAGAAGCCGCATAGATCGCCGTGAAGAGGAGGGGTCCGACGATCGAGGTCAGGCTGGTGAGCGCCGCCAGTGAGCCTTGCAGCTGCCCCTGACGTTCCTCATCCACCTGCCTGGACAACATTGCTTGCAGCGCCGGCATAAGCTTACTGTC
tetbP	251	24	0.9 962	119	ACTGTCAAGCTTGACTATCTATAAGGAAACACCTAAAGGTTTTGGAGCGTCAATAATGCATATGCAGGAAGACTTAAATCCATTTTGGGCGACAGTAGGCTTAGAAATAGAACCAGCAGGGAGAGGCGAAGGTCTTAGGTATATTTCTAATGTTTCAGTAGGGTCATTGCCAAAATCTTTTCAAAATGCAATTGAAGAAGCAGTTATTAAGACAAGTAAACAAGGATTATTTGGATGGGAAGCTTACTGTC
tetG	306	20	0.9 974	102	ACTGTCAAGCTTAGCCAATACTGGTTGAGGCCGCGAGCGACAAACCAACGGTCGCGGTGTTCCACTGAAAACGGTCCTCGCCATATATGACCCATAGGGCTGCAGGCACTTGGCCGATCAGTTGAATAATGAAGAAAACTGCGAAAAGCGCACCTAGCCCGCGCAATGCATCATCCAGCCGTAACAGAACGAATGGTTTGATGCGAACCGGCTTTCCGGTCCCGCCATGGCTGTGATGAGTCTCCTTGAGGAAAATGCAGGCAAGCAGGAACGCGATCCTTCTTCAACAATCAGAAGCTTACTGTC

tetH	251	24	0.9 943	110	ACTGTCAAGCTTTAAAAGGGGACCAATGATCCCGGTAATATTGGTTAGGCTCACCAGAGTACCTTGTAATTTCCCTTGCGCATTATCATCGACAGATTTTGATAAATAACCTTGTAATGCGGGTTGCCCCATACCTCCTGCCGCTAAGCAAATTAATGCTGGTAAGATGACCCAAACGTGGCCTATCCACGCTAATAATAAACAGCCCATCATATCAATAGACATACTGATCATAATGGAAGCTTACTGTC
tetL	251	24	0.9 989	108	ACTGTCAAGCTTTCCTTACTTATTATGGCTCGTTTTATTCAAGGGGCTGGTGCAGCTGCATTTCCAGCACTCGTAATGGTTGTAGTTGCGCGCTATATTCCAAAGGAAAATAGGGGTAAAGCATTTGGTCTTATTGGATCGATAGTAGCCATGGGAGAAGGAGTCGGTCCAGCGATTGGTGGAATGATAGCCCATTATATTCATTGGTCCTATCTTCTACTCATTCCTATGATAACAATAAGCTTACTGTC
tetM	251	24	0.9 957	116	ACTGTCAAGCTTAAACTACCTTAACAGAAAGCTTATTATATAACAGTGGAGCGATTACAGAATTAGGAAGCGTGGACAAAGGTACAACGAGGACGGATAATACGCTTTTAGAACGTCAGAGAGGAATTACAATTCAGACAGGAATAACCTCTTTTCAGTGGGAAAATACGAAGGTGAACATCATAGACACGCCAGGACATATGGATTTCTTAGCAGAAGTATATCGTTCATTATCAGTTAAGCTTACTGTC
tetO	251	24	0.9 982	110	ACTGTCAAGCTTGGAGTAAAATTACATGAAAATAATTAACTTAGGCATTCTGGCTCACGTTGACGCAGGAAAGACAACATTAACGGAAAGTTTATTGTATACCAGTGGTGCAATTGCAGAACTAGGGAGCGTAGATGAAGGCACAACAAGGACAGATACAATGAATTTGGAGCGTCAAAGGGGAATCACTATCCAGACAGCAGTGACATCTTTTCAGTGGGAGGATGTAAAAGTCAACAAAGCTTACTGTC
tetQ	251	24	0.9 985	126	ACTGTCAAGCTTTTGCAATAATGAGTGGTGTCATATTAAAGGGAAAGTTCCATTAAATACAAGTAAAGACTACGCCTCAGAAGTAAGTTCATACACTAAGGGCTTAGGCGTTTTTATGGTCAAGCCATGCGGGTATCAAATAACAAAAGGCGATTATTCTGATAATATCCGCATGAACGAAAAAGATAAACTTTTATTCATGTTCCAAAAATCAATGTCATCAAAATAATGGAGCGGTCAAGCTTACTGTC
tetT	251	24	0.9 972	115	ACTGTCAAGCTTTACAAAATAGATGCTAGATTTGGTGAATTGAAAACAATATATAAAGAACGACCTAAGAGAAACTCTAAAGCAGTAATCCATATAGAGGTTCCACCAAATCCTTATTGGGCATCTATTGGACTGTCAATAGAACCACTACCAATAGGGTCAGGATTATTATATAAGACAGAGGTGTCCTATGGATATTTAAATAATTCATTTCAAAATGCAGTAAAAGATGCTGTAGAAAGCTTACTGTC

tetW	251	24	0.9 895	122	ACTGTCAAGCTTTCTATGTCGGTATTTTCCTCCAGGACTATTTCCGGGGACAGCGACACCGTCTGCTTGATGATAATATCGGCGGAGAGCTTATCCCGAACAGACTGAACCACGCTCTGCAAATCAACGCCAGCCTGGTCGATCTTGTTGATAAAGATAACGGTGGGAATGTTCATTTTCCGCAGGGCATGGAACAGAATACGGGTCTGGGCCTGCACGCCATCTTTAGCGGAGATCACAAGCTTACTGTC
tetX	251	24	0.9 988	108	ACTGTCAAGCTTTTATTATTAGGATTCGCAAATAATAAATTACCTTGATGAGCAGCCATTAGCCGGTTTCCATTGCATAGCTGAAAAAATCCAGGACAGTTCACCTCTGGATGATGAATATCGGCTTGTATATTGAAAGTACCTGTTTCTTCAACTTCCGTGTCGGTAACAAATTTTCTTACTTTAGACATTCCACCATTGGCAATAATAACCAGATCTGCTGTTTCACTCGGTTTATTAAGCTTACTGTC

Table S4 – Number of biological and technical replicates for each sample type; total replicates in data output.

Sample Type	Day	Biological Replicates	Technical Replicates	Total Replicates
Manure	0	1	6	6
Manured soil	0	6	2	12
Manured effluent	11	6	2	12
Manured soil	17	3	2	6
Manured effluent	33	6	2	12
Manured effluent	61	6	2	12
Manured effluent	82	6	2	12
Manured soil	89	3	2	6
Manured effluent	110	6	2	12
Manured effluent	138	6	2	12
Manured soil	145	3	2	6

A

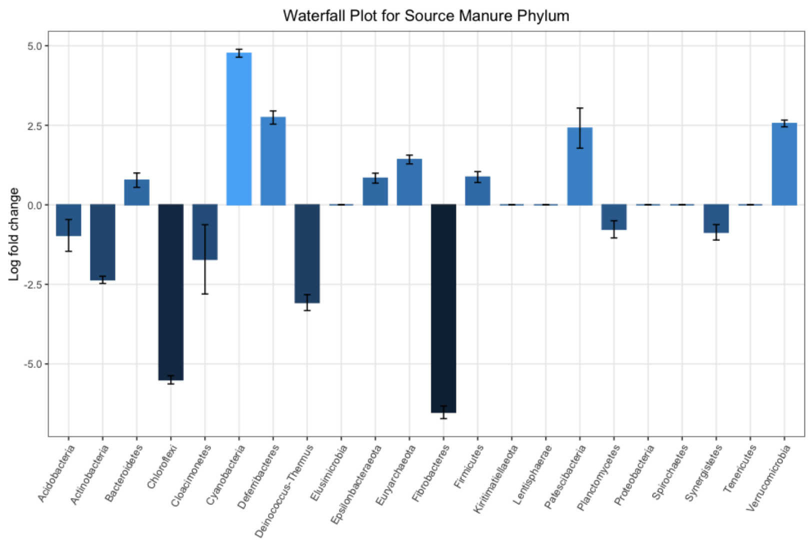
Waterfall Plot for Source Manure Phylum
Log fold change
5.0
2.5
0.0
-2.5
-5.0
Acidobacteria
Actinobacteria
Bacteroidetes
Chloroflexi
Cloacimonetes
Cyanobacteria
Deferribacteres
Deinococcus-Thermus
Elusimicrobia
Epsilonbacteraeota
Euryarchaeota
Fibrobacteres
Firmicutes
Kiritimatiellaeota
Lentisphaerae
Patescibacteria
Planctomycetes
Proteobacteria
Spirochaetes
Synergistetes
Tenericutes
Verrucomicrobia

B

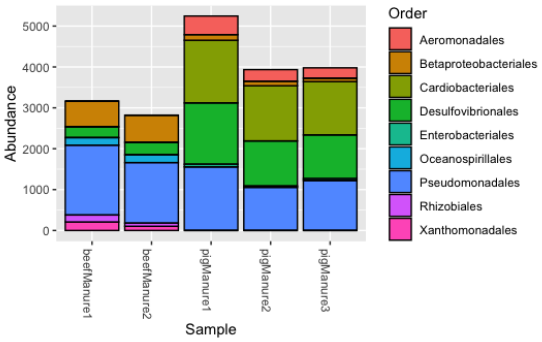
Abundance
5000
4000
3000
2000
1000
0
beefManure1
beefManure2
pigManure1
pigManure2
pigManure3
Sample
Order
Aeromonadales
Betaproteobacteriales
Cardiobacteriales
Desulfovibrionales
Enterobacteriales
Oceanospirillales
Pseudomonadales
Rhizobiales
Xanthomonadales

C

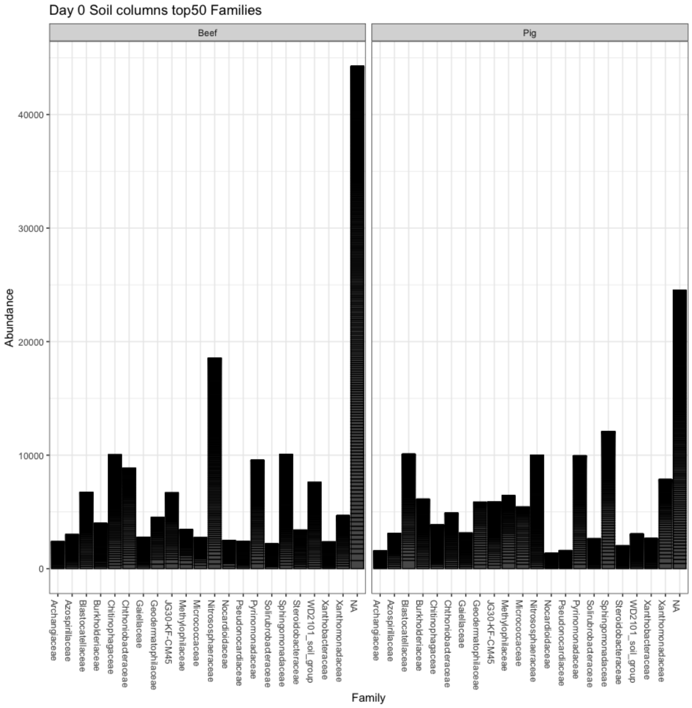
Day 0 Soil columns top50 Families
Beef
Pig
Abundance
40000
30000
20000
10000
0
Archangiaceae
Azospirillaceae
Blastocatellaceae
Burkholderiaceae
Chitinophagaceae
Chthoniobacteraceae
Gaiellaceae
Geodermatophilaceae
JG30-KF-CM45
Methylophilaceae
Micrococcaceae
Nitrososphaeraceae
Nocardioidaceae
Pseudonocardiaceae
Pyrinomonadaceae
Solirubrobacteraceae
Sphingomonadaceae
Steroidobacteraceae
WD2101_soil_group
Xanthobacteraceae
Xanthomonadaceae
NA
Family

Figure S2 – Taxa distribution of A) source manures at phylum level with swine dominated phyla on the positive y-axis and beef dominant phyla on the negative y-axis B) Proteobacteria within source manures at the Order level and C) top families from soil columns on day 0. For figure 5A, positive fold change is increased in swine manure while negative fold change is increased in beef manure.

Table S5 – ANCOM-BC analysis of effluent water from swine treated columns on phyla level taxa abundance over time. The effect of day was modeled using a cubic smoothing spline fit with day as a numeric factor and day-treatment as an interaction effect. Note that there is no day 0 for water treatment therefore the impact of manure decreases throughout the model (from day 11 onward). Only phyla with a minimum interaction p-value below 0.05 are included here.

Phylum	Treatment Manure	Intercept 1	Intercept 2	TreatmentManure :Intercept1	TreatmentManure :Intercept2	min interaction p-val
PAUC34f	1	0	0	0	0	0.00E+00
Deinococcus-Thermus	8.52E-80	0.02577422	0.01911378	8.23E-22	1	8.23E-22
Tenericutes	2.10E-19	0.50005738	0.00199061 5	4.60E-15	1	4.60E-15
Bacteroidetes	1.11E-21	1	1	2.78E-06	1	2.78E-06
Firmicutes	1.82E-08	0.019807551	0.00031526	0.000784008	1	7.84E-04
Acidobacteria	1.70E-13	0.003554933	1	0.001027478	1	1.03E-03
Elusimicrobia	1.44E-09	1	1	1	0.002322543	2.32E-03
Chloroflexi	1	1	3.66E-05	1	0.003071264	3.07E-03
Fibrobacteres	0.1065099 85	0.00322436	0.28096722	0.004678007	1	4.68E-03
Nanoarchaeae ota	1	0.004154535	0.37018976	1	0.00635071	6.35E-03
Euryarchaeota	3.09E-07	0.382587011	1	1	0.047614569	4.76E-02

Table S6 – ANCOM-BC analysis of soil from swine treated columns on phyla level taxa abundance over time. The effect of day was modeled using a cubic smoothing spline fit with day as a numeric factor and day-treatment as an interaction effect. Note that the model includes day 0 soil samples (prior to manure treatment).

Phylum	TreatmentManu re	Interaction1	Interaction2	TreatmentManure :1	TreatmentManur e:2	min interaction p-val
Spirochaetes	0.041422733	0	0	0	0	0.00E+00
FCPU426	1	0	0	0	0	0.00E+00
Patescibacteria	0.175298961	1	0.081273074	4.70E-08	0.006395614	4.70E-08
Proteobacteria	0.071197795	0.252339743	1	1.81E-05	0.375233909	1.81E-05

Chlamydiae	1	1	1	1.86E-05	1	1.86E-05
FBP	1	8.29E-08	0.816565837	2.08E-05	0.000979861	2.08E-05
Gemmatimonadetes	1	0.010432919	1	0.000455716	0.375233909	4.56E-04
Verrucomicrobia	1	1	1	1	0.000531525	5.32E-04
Latescibacteria	1	0.001184832	0.067765976	1	0.0013258	1.33E-03
WPS-2	1	0.003424369	1	0.004323307	0.050942116	4.32E-03
Rokubacteria	0.678048083	0.016159741	1	1	0.010482981	1.05E-02
Thaumarchaeota	1	1	1	1	0.036654421	3.67E-02
Elusimicrobia	1	1	1	1	0.050942116	5.09E-02
Tenericutes	1	1	0.193016652	0.18842715	1	1.88E-01
Planctomycetes	1	1	1	0.210789292	1	2.11E-01
Euryarchaeota	0.019005776	0.153990273	1	1	0.296997565	2.97E-01
Bacteroidetes	0.111460012	0.396949924	1	0.309430892	1	3.09E-01
Actinobacteria	0.776104714	1	1	0.325694466	1	3.26E-01
Fibrobacteres	1	0.66411432	0.458772809	1	0.362760983	3.63E-01
Cyanobacteria	1	0.394472212	0.134150166	1	0.462556875	4.63E-01
Hydrogenedentes	1	0.163015179	1	0.573662926	1	5.74E-01
Chloroflexi	1	1	1	1	0.578579798	5.79E-01
WS2	0.678048083	0.005425976	0.24119559	1	0.578579798	5.79E-01
Nanoarchaeaeota	1	1	1	1	0.752766718	7.53E-01
Acidobacteria	1	1	1	1	1	1.00E+00
Firmicutes	0.000460528	1	1	1	1	1.00E+00
Entotheonellaeota	1	1	1	1	1	1.00E+00
Nitrospirae	1	1	1	1	1	1.00E+00
Armatimonadetes	1	0.285538541	1	1	1	1.00E+00
Dependentiae	1	1	1	1	1	1.00E+00
Omnitrophicaeota	1	1	1	1	1	1.00E+00
BRC1	1	0.66411432	1	1	1	1.00E+00

Table S7 – ANCOM-BC analysis of effluent water from beef treated columns on phyla level taxa abundance over time. The effect of day was modeled using a cubic smoothing spline fit with day as a numeric factor and day-treatment as an interaction effect. Note that there is no day 0 for water treatment therefore the impact of manure decreases throughout the model (from day 11 onward). Only phyla with a minimum interaction p-value below 0.05 are included here.

**Phylum**	**TreatmentManure**	**Interaction1**	**Interaction2**	**Treatment Manure:X1**	**Treatment Manure:X2**	**min interaction p-val**
PAUC34f	1	0	0	0	0	0.00E+00

Deinococcus-Thermus	8.52E-80	0.02577422	0.019114	8.23E-22	1	8.23E-22
Tenericutes	2.10E-19	0.50005738	0.001991	4.60E-15	1	4.60E-15
Bacteroidetes	1.11E-21	1	1	2.78E-06	1	2.78E-06
Firmicutes	1.82E-08	0.01980755	0.000315	0.000784008	1	7.84E-04
Acidobacteria	1.70E-13	0.00355493	1	0.001027478	1	1.03E-03
Elusimicrobia	1.44E-09	1	1	1	0.002322543	2.32E-03
Chloroflexi	1	1	3.66E-05	1	0.003071264	3.07E-03
Fibrobacteres	0.106509985	0.00322436	0.280967	0.004678007	1	4.68E-03
Nanoarchaeaeota	1	0.00415454	0.37019	1	0.00635071	6.35E-03
Euryarchaeota	3.09E-07	0.38258701	1	1	0.047614569	4.76E-02
Actinobacteria	0.008135353	0.04907734	1	0.095800762	1	9.58E-02
Gemmatimonadetes	0.000129301	0.38258701	1	0.20466408	1	2.05E-01
Cyanobacteria	0.128591472	1	0.37019	0.20466408	0.715417206	2.05E-01
Thaumarchaeota	0.06815344	0.09360944	0.002052	1	0.313353823	3.13E-01
Omnitrophicaeota	0.011273978	0.08908513	1	1	0.313353823	3.13E-01
FCPU426	9.53E-06	0.10979793	1	1	0.313353823	3.13E-01
Verrucomicrobia	3.00E-08	0.01244704	1	0.706763393	0.39047095	3.90E-01
Lentisphaerae	1	1	0.068873	1	0.667723545	6.68E-01
Spirochaetes	0.000236027	3.55E-05	0.001239	1	0.739631613	7.40E-01
WPS-2	1	1	1	0.911357918	0.763199157	7.63E-01
Proteobacteria	1	1.32E-07	0.2315	0.911357918	1	9.11E-01
Latescibacteria	1	0.42609807	0.481585	1	1	1.00E+00
Planctomycetes	1	1	1	1	1	1.00E+00
Nitrospirae	0.844457153	0.38258701	1	1	1	1.00E+00
Rokubacteria	0.09433629	0.39565209	1	1	1	1.00E+00
Dependentiae	1	1	0.452535	1	1	1.00E+00
Hydrogenedentes	1	1	1	1	1	1.00E+00
Patescibacteria	1.01E-07	1	1	1	1	1.00E+00
Armatimonadetes	0.472207119	0.0306566	1	1	1	1.00E+00
WS2	1	0.27776406	1	1	1	1.00E+00
BRC1	1	1	1	1	1	1.00E+00
Diapherotrites	1	0.01215734	1	1	1	1.00E+00

Chlamydiae	0.162488232	1	1	1	1	1.00E+00
Margulisbacteria	0.616726762	0.7703518	0.413459	1	1	1.00E+00
FBP	1	0.7703518	1	1	1	1.00E+00

Figure S3 – Abundance of 5 key phyla in soil from cattle-manure treated columns over time. The effect of day was modeled using a cubic smoothing spline fit with day as a numeric factor and day-treatment as an interaction effect and log abundance was calculated using ANCOM-BC. Note that the model includes day 0 soil samples (prior to manure treatment).

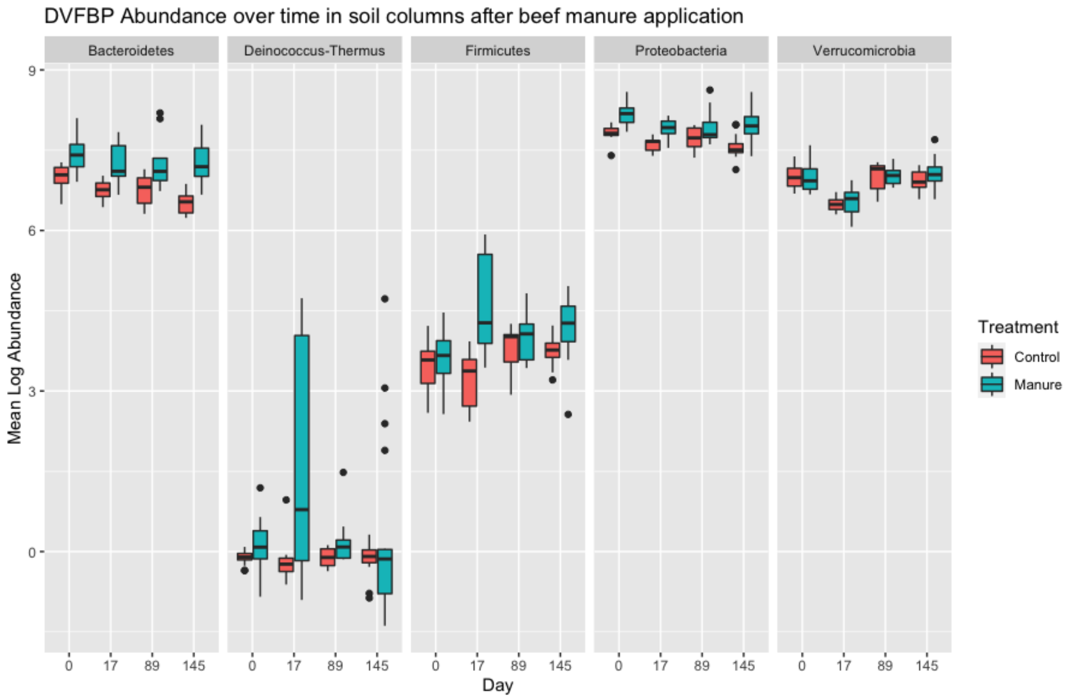

Table S8 –Mann-Whitney U-test p-values for significant differences in median abundance of ARGs between swine and beef manure sample types, combined control samples, and manure-treated.

	p-value
Manure Samples	5.34E-05
Control Samples	0.968
Manure-treated Samples	1.80E-11

Table S9 – Mann-Whitney U-test p-values for differences in median abundance of ARGs in swine or beef manure-treated samples and paired controls.

Gene	Swine Water Day 11	Swine Water Day 33	Swine Water Day 61	Swine Water Day 110	Swine Water Day 138	Swine Soil Day 17	Swine Soil Day 89	Swine Soil Day 145	Beef Water Day 11	Beef Water Day 33	Beef Water Day 61	Beef Water Day 110	Beef Water Day 138	Beef Soil Day 17	Beef Soil Day 89	Beef Soil Day 145
tetX	0.00615061	NA	0.40465676	NA	NA	0.063603	NA	NA	0.0027784	0.4046568	0.07401	NA	NA	0.19671	NA	NA
tetW	0.00277843	NA	NA	NA	NA	0.196706	NA	NA	0.1757343	NA	NA	NA	NA	NA	NA	0.40466
tetT	0.00277843	NA	NA	NA	NA	0.063603	NA	NA	0.4046568	NA	NA	NA	NA	0.50499	NA	NA
tetQ	0.00277843	0.17573434	NA	NA	NA	0.063603	0.196706	0.404657	0.1757343	0.4046568	NA	NA	NA	0.50499	NA	NA
tetO	0.00366455	0.07400997	0.40465676	NA	NA	0.063603	NA	NA	0.4046568	0.4046568	NA	NA	NA	NA	NA	NA
tetM	0.00366455	0.02844066	NA	NA	NA	0.063603	NA	0.404657	0.0096217	0.4046568	NA	NA	NA	0.50499	NA	NA
tetL	0.00277843	NA	NA	NA	NA	0.063603	NA	NA	NA	NA	NA	NA	NA	NA	NA	NA
tetH	0.00277843	NA	NA	NA	NA	0.063603	NA	NA	0.0027784	NA	NA	NA	NA	NA	NA	NA
tetG	0.34075447	NA	0.65530639	0.02844066	0.18228718	NA	0.196706	0.404657	0.0027784	0.0608077	0.008658	0.7525538	0.1797125	0.50499	1	0.40466
tetbP	0.07400997	NA	NA	NA	NA	NA	NA	NA	0.07401	NA	0.4046568	NA	NA	NA	0.50499	NA
tetA	NA	NA	1	NA	NA	NA	NA	NA	NA	0.4046568	NA	NA	NA	NA	NA	NA
tet44	0.00277843	0.40465676	0.40465676	NA	NA	0.196706	0.504985	0.404657	0.4046568	NA	NA	NA	NA	NA	NA	NA
tet(36)	0.00277843	0.40465676	NA	NA	NA	0.063603	0.504985	NA	0.4046568	NA	NA	NA	NA	0.50499	NA	NA
tet(33)	0.57247557	0.00277843	0.93722944	0.03794147	0.30952381	1	0.7	0.280475	0.1805029	0.0047718	0.008658	0.3751171	0.1797125	0.50656	0.4	0.57248
tet(32)	0.00962172	NA	NA	NA	NA	NA	NA	NA	NA	NA	NA	NA	NA	NA	NA	NA
sul2	0.00277843	0.02844066	0.40465676	NA	NA	0.063603	NA	0.404657	0.0027784	0.4619505	0.1757343	0.4046568	NA	0.50499	NA	0.17573
sul1	0.00277843	0.07400997	0.40465676	0.40465676	NA	0.196706	NA	NA	0.0027784	1	NA	NA	NA	0.50499	NA	NA
strB	0.00277843	0.40465676	0.40465676	NA	NA	0.063603	NA	NA	0.0027784	0.07401	0.0284407	0.4046568	NA	0.50499	NA	0.40466
strA	0.17573434	NA	NA	NA	NA	0.504985	NA	NA	0.1757343	NA	NA	NA	NA	NA	NA	NA
sat4	0.00277843	NA	NA	NA	NA	0.063603	NA	NA	NA	0.4046568	NA	NA	NA	NA	NA	NA
mefA	0.02844066	NA	NA	NA	NA	0.504985	NA	NA	NA	0.4046568	NA	NA	NA	0.50499	NA	NA
mef(B)	0.00962172	NA	NA	NA	NA	0.504985	NA	NA	NA	NA	NA	NA	NA	NA	NA	NA
lnuC	0.02844066	NA	NA	NA	NA	0.063603	NA	NA	NA	NA	NA	NA	NA	NA	NA	NA
lnuA	0.00277843	NA	NA	NA	NA	0.504985	NA	NA	0.0284407	NA	NA	NA	NA	0.50499	NA	NA
lnu(F)	0.00277843	NA	NA	NA	NA	0.063603	NA	NA	0.4046568	NA	0.4046568	NA	NA	NA	NA	NA
ermX	0.17573434	NA	NA	NA	NA	NA	NA	NA	NA	NA	NA	NA	NA	NA	NA	NA
ermT	0.00962172	NA	NA	NA	NA	0.196706	NA	NA	NA	NA	NA	NA	NA	NA	NA	NA
ermA/ermTR	0.17573434	NA	NA	NA	NA	NA	NA	NA	NA	NA	NA	NA	NA	NA	NA	NA
erm(F)	0.00962172	NA	NA	NA	NA	0.063603	NA	NA	0.4046568	NA	NA	NA	NA	NA	NA	NA
erm(C)	0.02844066	NA	NA	NA	NA	NA	NA	NA	NA	NA	NA	NA	NA	NA	NA	NA
erm(B)	0.00277843	0.07400997	NA	NA	NA	0.063603	NA	NA	NA	0.4046568	NA	NA	NA	NA	NA	NA
erm(35)	0.00277843	NA	0.40465676	NA	NA	0.063603	NA	NA	NA	NA	NA	NA	NA	NA	NA	NA
aph(3)-ia	0.00962172	NA	NA	NA	NA	0.063603	NA	NA	NA	NA	NA	NA	NA	NA	NA	NA
ant6-ib	0.00277843	0.17573434	NA	NA	NA	0.076523	0.504985	NA	NA	NA	NA	NA	NA	NA	NA	NA
ant6-ia	0.00962172	NA	NA	NA	NA	0.063603	NA	NA	NA	NA	NA	NA	NA	NA	NA	NA
aadA9	0.02844066	0.17573434	0.40465676	NA	NA	NA	NA	0.404657	0.0284407	NA	0.4046568	NA	NA	NA	NA	0.40466
IS6100	0.02499206	0.24578549	0.79764787	0.40465676	0.32605752	1	1	0.175734	0.2215095	0.5611487	1	1	1	0.50499	NA	0.40466
IS26	0.06085645	NA	NA	NA	NA	0.063603	NA	NA	0.0284407	NA	NA	NA	NA	NA	NA	NA
intl3	0.93607468	0.00366455	0.93722944	0.02499206	0.30952381	0.4	0.7	0.872559	0.470393	0.0021645	1	0.2945516	0.525809	0.50499	0.1	0.48485
intl2	0.02844066	NA	NA	NA	NA	0.063603	NA	NA	0.0027784	NA	NA	NA	NA	NA	NA	NA
intl1	0.07400997	0.12907261	0.74834857	0.00277843	0.24578549	NA	0.184039	0.404657	0.1757343	0.07401	0.3939394	0.1757343	0.1797125	NA	0.18404	0.54967
IncP_oriT	0.40465676	0.40465676	0.40465676	0.40465676	0.59926596	0.504985	0.504985	0.404657	0.2457855	NA	0.4046568	0.07401	NA	NA	NA	NA
